# A Forward Genetics Strategy for High‐Throughput Gene Identification via Precise Image‐Based Phenotyping of an Indexed EMS Mutant Library

**DOI:** 10.1002/advs.202514793

**Published:** 2025-09-29

**Authors:** Haojie Wang, Fujun Sun, Zeyu Shi, Yufeng Yang, Yi Ding, Tengteng Zhang, Caihong Zhao, Jingzhong Xie, Yaqian Zhang, Caihua Li, Wenqiang Tang, Junming Li, Xigang Liu, Shusong Zheng, Ni Jiang, Fei He, Shuzhi Zheng

**Affiliations:** ^1^ Ministry of Education Key Laboratory of Molecular and Cellular Biology Hebei Collaboration Innovation Center for Cell Signaling and Environmental Adaptation Hebei Key Laboratory of Molecular and Cellular Biology College of Life Sciences Hebei Normal University Shijiazhuang 050024 China; ^2^ Laboratory of Advanced Breeding Technologies Institute of Genetics and Developmental Biology Chinese Academy of Sciences Beijing 100101 China; ^3^ University of Chinese Academy of Sciences Beijing 100049 China; ^4^ Centre of Excellence for Plant and Microbial Science (CEPAMS) JIC‐CAS Beijing 100101 China; ^5^ Shijiazhuang Academy of Agriculture and Forestry Science Shijiazhuang 050024 China

**Keywords:** gene‐based association, genetic resources, indexed EMS library, precise phenotyping

## Abstract

Ethyl methanesulfonate (EMS) mutants are widely used for genetic analysis; however, EMS‐derived mutant populations are not amenable to traditional genome‐wide association studies (GWAS) because the EMS mutations are present at extremely low frequencies. To address this challenge, this work develops the GeneHunter‐Gene‐Level Association (GH‐GLA) pipeline using an EMS‐generated population of wheat (*Triticum aestivum*) mutants and an image‐based phenotyping platform. GH‐GLA enables comprehensive exploration of phenotypic variation induced by genome‐wide saturation mutagenesis. Using GH‐GLA to quantify 83 traits in the wheat population reveals that variation in spikelet geometry is significantly associated with key agronomic traits, including thousand‐kernel weight. Using this indexed wheat EMS population and phenotype data, GH‐GLA identified 5905 genes that are significantly associated with specific traits. Analysis of knockouts generated by gene editing, together with haplotypes affected by selection during breeding and genetic variation in 262 wheat accessions, confirm the roles of *TaAN‐1*, *TaBAM5L*, and *TaXTH28L* in regulating thousand‐kernel weight and spikelet angle. Furthermore, this work establishes an epistatic interaction network between gene pairs to elucidate their combined effects on the phenotype. Overall, GH‐GLA provides a powerful strategy for functional gene identification, and the alleles discovered here offer valuable genetic resources for crop improvement.

## Graphical abstract

The GeneHunter‐Gene‐Level Association (GH‐GLA) pipeline enables high‐throughput gene identification in an indexed EMS population of wheat cultivar KN9204. It identifies 5905 trait‐associated wheat genes and validates key regulators of kernel weight and spikelet architecture via gene editing and haplotype analysis.

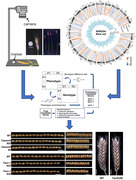

## 1 Introduction

Mutants generated by ethyl methanesulfonate (EMS) have uses in germplasm innovation, development of new varieties, and gene functional research.^[^ ^1^, ^2^, ^3^ ^]^ In addition, EMS mutant populations have theoretical advantages for the genetic dissection of traits, including minimal population structure and high genetic diversity. However, technical issues limit realization of the full potential of EMS mutagenesis for gene cloning. For example, traditional genetic mapping has low resolution, typically locating candidate genes to megabase‐scale intervals and necessitating labor‐intensive fine‐mapping.^[^ ^4^ ^]^ Furthermore, low‐throughput conventional mapping impedes the systematic discovery of genes in large mutant libraries. The random distribution and sparse density of EMS‐induced mutations result in extremely low minor allele frequencies (MAF), typically below 0.01 across the entire population at individual loci. This sparsity severely limits the effectiveness of conventional genome‐wide association studies (GWAS). In addition, we lack robust analytical tools and high‐resolution mapping strategies.^[^ ^5^ ^]^

The expansion in the scale and diversity of EMS mutant libraries has exacerbated these challenges, and many EMS mutant libraries have been established. For instance, Lu et al. constructed a near‐saturation indexed mutant library in the maize (*Zea mays*) inbred line B73,^[^ ^6^ ^]^ and Lian et al. developed an indexed mutant library for cotton (*Gossypium hirsutum*).^[^ ^7^ ^]^ In wheat (*Triticum aestivum*), Krasileva et al. generated an indexed mutant library.^[^ ^8^ ^]^ These mutant collections have been successfully used to link genotypes to agronomic traits. For example, Xiong et al. mapped a mutation in a gene that is responsible for a yellow‐green leaf phenotype in a wheat mutant library generated in the Jing411 background through trait‐based segregation analysis.^[^ ^9^ ^]^

Traditional phenotyping and genetic mapping are constrained by high labor requirements, subjective bias, and high costs.^[^ ^10^, ^11^ ^]^ Phenomics addresses these constraints by using nondestructive, high‐throughput, and quantitative measurements of multiple traits.^[^ ^12^, ^13^ ^]^ Recent advances in deep learning have further enhanced image‐based phenotyping, allowing the automated extraction of color, texture, and structural features from plant images.^[^ ^14^ ^]^ The high‐throughput analysis of image‐based traits (referred to hereafter as i‐traits) decomposes complex phenotypes into quantifiable subtraits, thereby improving the precision of genetic dissection.^[^ ^15^ ^]^

High‐throughput phenotyping platforms may enable the rapid and efficient acquisition of diverse phenotypic data across large populations to dissect important agronomic traits such as grain yield. Thousand‐kernel weight (TKW), grain number per spike (GN), and number of spikes per area are primary determinants of final yield in wheat.^[^ ^16^ ^]^ Modifications in spike architecture can enhance GN and TKW, thereby increasing grain yield.^[^ ^17^, ^18^ ^]^ Spike morphology is primarily governed by the arrangement and structure of spikelets, the fundamental units of the spike that contain florets, glumes, and the lemma. The shape of each spike reflects the spikelet angle, spikelet gap, and spikelet length.^[^ ^19^ ^]^ These geometric traits affect spike morphology and thus influence yield. The genetic dissection of spike morphology traits and their regulatory networks is critical for the development of high‐yielding wheat varieties.

In this study, we integrated high‐throughput image‐based phenotyping with large‐scale genotyping data from the KN9204 wheat indexed EMS population^[^ ^20^ ^]^ and developed an R‐based tool, GeneHunter based on Gene‐Level Association (GH‐GLA), specifically tailored for EMS populations. GH‐GLA enables high‐throughput genotype–phenotype association studies by directly identifying functional candidate genes, thereby circumventing the broad‐interval mapping problem typical of traditional GWAS. Using GH‐GLA, we identified candidate genes associated with 83 traits and used gene editing to validate the roles of TaAN‐1 and TaBAM5L in regulating TKW. Collectively, our results indicate that GH‐GLA offers a scalable and generalizable tool that can be applied across crops with available indexed EMS libraries. By integrating phenomics and genomics in EMS populations, our study provides a robust methodology for dissecting complex traits, particularly in polyploid species.

## 2 Results

### 2.1 Phenomics Technology Enabled Rapid Acquisition of 83 Traits

**Figure 1:**
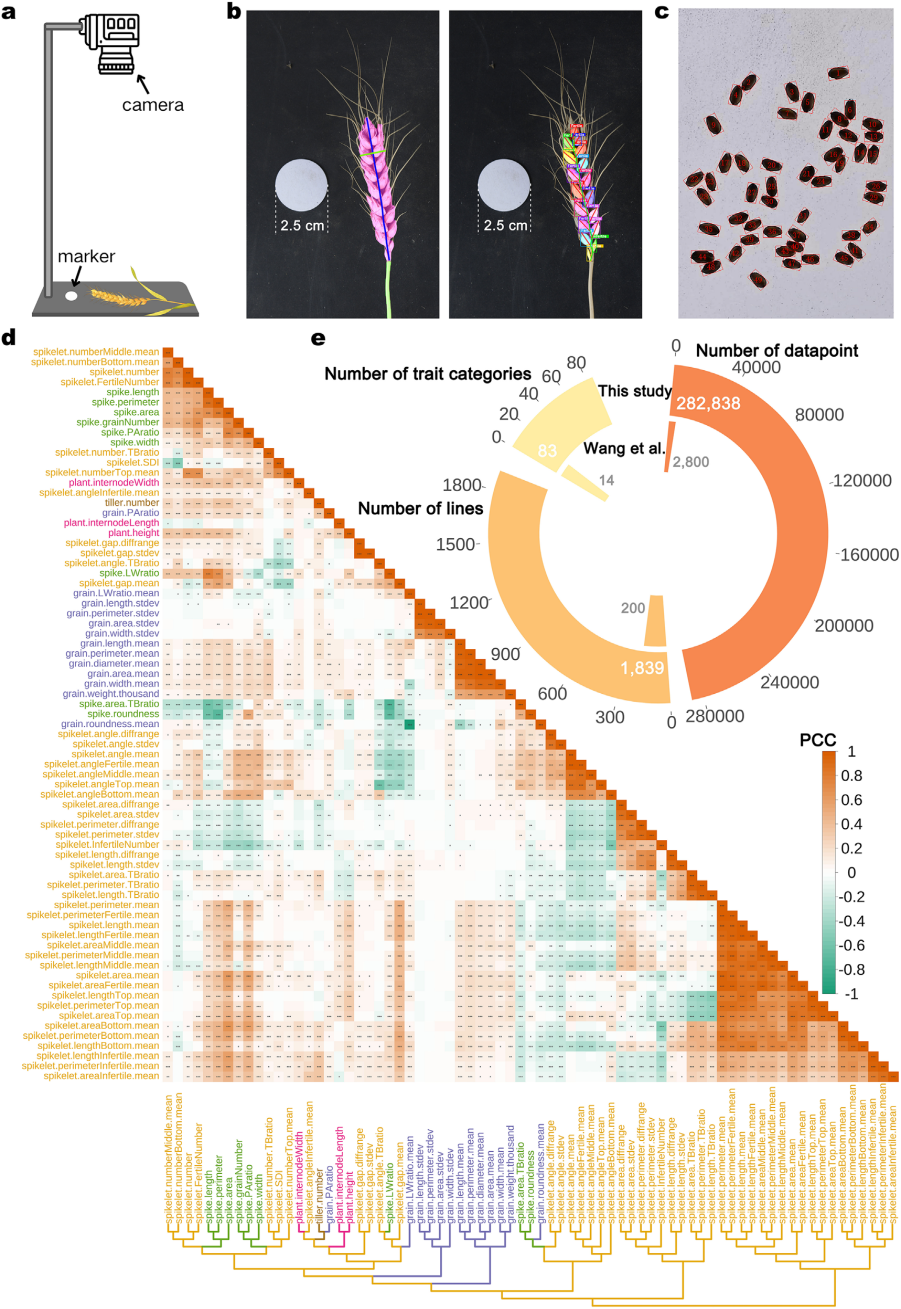
Pipeline for phenotype extraction and analysis of phenomics data. a) Diagram illustrating the phenotyping collection platform. b) Phenotypic identification based on deep learning and image processing: wheat spikes are phenotyped by extracting masks and the skeleton after segmentation (left); spikelets are phenotyped using instance segmentation (right). c) Identification of grain traits using a seed analyzer. d) Heatmap illustrating the Pearson's correlation coefficients between pairs of wheat traits. The color gradient represents the strength and direction of the correlation (***, *p* < 0.001; **, *p* < 0.01; *, *p* < 0.05). The dendrogram at the bottom indicates the hierarchical clustering of traits based on their correlation patterns. Colors in the dendrogram distinguish different phenotypic categories. e) Pie chart comparing three key metrics collected in this study and from Wang et al. (2023): number of phenotypic trait categories, mutant lines analyzed, and total phenotyping data points.

We previously generated an indexed EMS mutation library in the common wheat accession KN9204,^[^ ^20^ ^]^ of which only 200 lines were phenotyped for a set of 12 agronomic traits as a proof of concept. To obtain a comprehensive phenotype dataset from the EMS mutant population, we established an image‐based phenotyping pipeline in which intact whole plants or spikes from each line are placed on a platform with a scale marker for standardized imaging (**Figure** **1**a). We captured 20 963 raw images (18 388 spike images and 2575 whole‐plant images). We then performed a deep learning–based image analysis to extract plant architecture traits, spike traits, and spikelet traits from these images (Figure 1b, Figure S1a, Supporting Information). Additionally, we obtained grain traits by averaging values extracted from the photographs using a seed analyzer (Figure 1c). This pipeline enabled the high‐throughput acquisition of i‐traits for the entire EMS population and its wild‐type controls. We extracted five major categories of traits—traits related to plant architecture, grains, tillers, spikes, and spikelets from 1839 EMS‐induced mutant lines in the KN9204 background (Table S1, Supporting Information), representing 282 838 data points for 83 traits (Figure 1e). This is 101 times more data points than previously collected for the first 200 lines (200 lines × 12 traits = 2400 data points). This substantial expansion in measured traits and processed population size highlights the power of deep learning–based image analysis as an efficient, rapid, and accurate phenotyping tool, particularly well‐suited for large‐scale populations.

As the i‐traits measured in this study have not been previously explored, we looked for correlations among different phenotypes by calculating Pearson's correlation coefficients between pairs of traits. All traits (classical traits and i‐traits) could be clustered into various modules. Notably, TKW showed a strong positive correlation with traits such as grain length (GL), grain width (GW), grain perimeter, grain area, and grain diameter (Figure 1d). We identified a distinct positive correlation forming a module comprising different spike traits (GN, spike width, spike length, spike area, spike perimeter, and the number of fertile spikelets), suggesting a tight relationship between these traits. Similarly, we observed a clear positive correlation between the spike length‐to‐width ratio (spike.LWratio) and spike perimeter, which is consistent with basic geometric principles. In contrast, the number of tillers exhibited significant negative correlations with grain roundness and spike roundness, suggesting a potential trade‐off between vegetative branching and reproductive organ morphology (Figure 1d, Table S2, Supporting Information).

These results demonstrate that we have developed a high‐efficiency phenotyping method based on deep learning–driven image analysis tools, which allowed us to perform an in‐depth exploration of the phenotypic variation caused by induced mutations with potential applications for association studies.

### 2.2 EMS Mutagenesis Expands the Phenotypic Range of Wheat Cultivars

**Figure 2:**
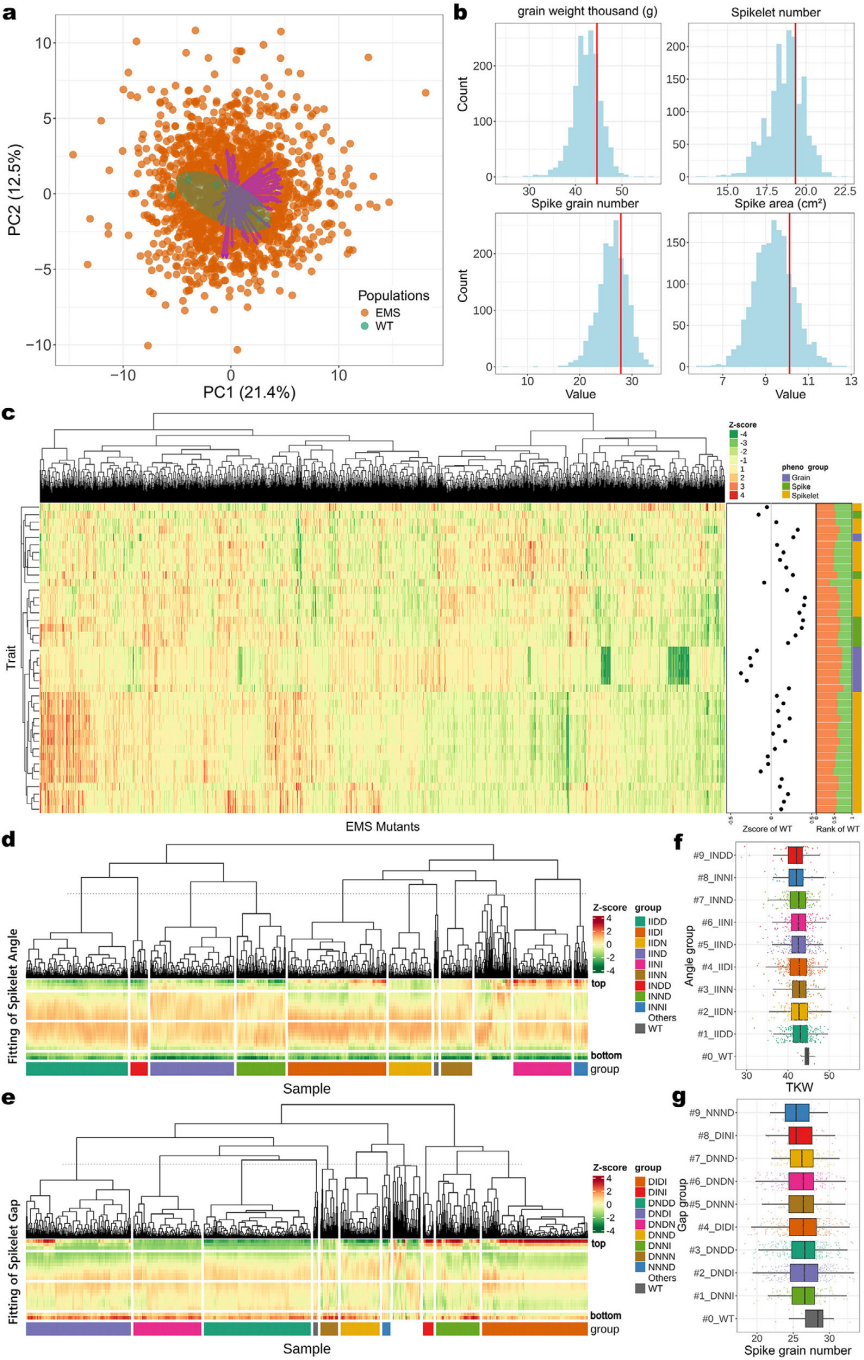
Phenomics analysis and quantification of geometric phenotypes. a) Principal component analysis (PCA) plot of phenotypic variation between EMS (orange) and wild type (WT; green) populations (PC1 = 21.4%, PC2 = 12.5%), demonstrating clear separation between the mutant and wild‐type groups. b) Distributions of phenotypic data collected from the EMS population. The red lines indicate the average value of the wild‐type population; note that most mutant samples have values that are left‐shifted relative to the WT mean. c) Clustering of 41 phenotypes from the EMS population after *Z*‐score normalization. The *Z*‐scored average values for the WT, along with the ranking of the WT population within the EMS population, are shown to the right of the heatmap. Color‐coded phenotypic categories are shown in the rightmost bar: grain‐related traits (purple), spike characteristics (green), and spikelet features (yellow). d,e) Clustering of fitted values for spikelet angle (d) and spikelet gap (e) in the EMS population, categorized by group. f,g) Boxplots of the agronomic traits TKW and grain number per spike across subgroups classified based on the spikelet geometric parameters spikelet angle (f) and spikelet gap (g), ordered by ascending mean trait value (dark gray: WT subgroup). Each box plot represents the median (center line), interquartile range (box), and minimum/maximum values excluding outliers (whiskers). Outliers are shown as individual dots.

We planted wild‐type KN9204 plants along with the EMS panel at 15 random locations in the experimental field. Therefore, the observed variability within the wild‐type plants may reflect changes in the adaptability of KN9204 to subtle variations in the field environment. When we performed a principal component analysis of the EMS and wild‐type populations, using all phenotypic values, the resulting principal component analysis (PCA) plot showed a more dispersed phenotypic distribution in the EMS population than in the wild type. The EMS lines radiated outward in all directions from the center of the plot, whereas the wild‐type plants exhibited no clear directional distribution (**Figure** **2**a), as confirmed by Permutational Multivariate Analysis of Variance (PERMANOVA) testing (*P*‐value = 0.012), thus demonstrating that the range of phenotypes was due to EMS mutations. Thus, the EMS population, with many random and diverse phenotypes not present in the wild type or natural populations, could be a valuable resource for breeding (Figure S2a, Supporting Information).

In addition to artificially increasing genetic diversity, EMS mutagenesis often disrupts genetic stability,^[^ ^21^, ^22^ ^]^ resulting in inferior traits in mutants compared to the wild type (Figure 2b, Figure S2b, Table S3, Supporting Information). We hypothesized that a phenomics exploration of the population would help us harness the phenotypic potential of EMS populations. We therefore normalized 41 representative phenotypes across the entire population using *Z*‐scores and then performed a clustering analysis, leading to the classification of the population into distinct categories based on the phenotypic profile of each EMS line (Figure 2c). We speculated that, at the individual plant level, phenotypic changes induced by EMS mutagenesis are random. As a constrained system, each individual mutant plant shows a limited range of phenotypic changes. When we shuffled the order of lines for each phenotype, we did not identify any discernible clustering patterns (Figure S2c, Supporting Information). Additionally, the peak in the distribution of most phenotypic values was below the average value for the wild type (Figure 2c, Table S3, Supporting Information). The average values for these 41 phenotypic traits across the EMS population ranged from −4.04% lower to 4.60% higher than those in the wild type; 30 traits had higher average values than the wild‐type average, and 11 traits had lower average values (Figure 2c). Relative to the wild‐type averages, the phenotypic trait with the largest drop in average value was the number of grains per spike (spike.grainNumber), whereas the trait with the largest increase was the angle of infertile spikelets (spikelet.angleInfertile.mean). Although the overall phenotypic values across the EMS population were lower than in the wild type, individual plants did display greater phenotypic values for some traits. For example, 67 EMS lines had TKW values exceeding 120% the wild‐type average (Figure 2c). We conclude that our large‐scale phenomics study of the EMS population provides insights into the potential phenotypic combinations of individual plants, which will help us narrow down the selection range for ideal plant types.

### 2.3 Large‐Scale EMS Mutations Generate Limited Types of Spike Morphology

We focused on features of spikelet morphology derived from i‐traits and extracted the geometric values for each spikelet segment, including spikelet angle and gap. The wild‐type KN9204 plants produced an average of 19.2 spikelets in the field, with the mean spikelet angles for each plant ranging from 30.7° to 37.7° and the mean spikelet gap ranging from 0.37 mm to 0.48 mm. In the KN9204 EMS population, the plants had an average of 18.7 spikelets, with spikelet angles ranging from 17.0° to 52.0° and a spikelet gap between 0.30 mm and 0.59 mm (Table S3, Supporting Information).

Since the number of spikelets differed across lines, we applied a fourth‐order polynomial fit to the spikelet data for each line and scaled the data to the [0, 20] range (with 0 being the bottom and 20 being the top of the spike). This approach ensured that the spikelet angle of each line follows a standardized trajectory curve. We then interpolated these standardized data and performed a clustering analysis (Figure 2d). When the distribution of spikelet angle was used as a phenotype, the entire EMS population clustered into nine major distinct groups. When the spikelet angle values were randomly assigned to EMS lines, no clear clustering pattern emerged (Figure S2d, Supporting Information). In the heatmap, red and green areas represent spikelet angles larger and smaller than the population mean, respectively (Figure 2d). We observed greater variation in spikelet angle at the base and top of the spike than in the middle section. In wild‐type plants, the ranges of spikelet angle at the bottom, lower‐middle, and upper‐middle, were 13.3 to 28.3°, 33.3 to 43.6°, 28.4 to 42.65°, and 13.6 to 53.6°, respectively. However, in the EMS population, the spikelet angle range was 5.5 to 38.9°, 19.7 to 54.9°, 17.3 to 55.1°, and 8.0 to 71.5° at the corresponding positions, respectively (Figure 2d). The greater values seen for the EMS population reflect the phenotypic potential induced by random mutations.

To help define the type of change for the entire spike, we calculated the average value for each part in the EMS population and categorized each spike part as increased (I), unchanged (N), or decreased (D) when the average value was greater than, approximately equal to, or less than the population average, respectively. There are 81 possible combinations (3 × 3 × 3 × 3), although some were rarely seen (e.g., DDDD). We determined that spikelet angle in the KN9204 EMS mutant population fell mainly into the IDD, IIND, and IIDI types, which accounted for 19.2%, 18.5%, and 15.8% of the population, respectively. The spikelet angles for the IIDD, IIND, and IIDI types were as follows: in the bottom section of the spike, the ranges in angle were 5.5 to 38.9°, 13.2 to 23.8°, and 10.2 to 37.1°, respectively; in the lower‐middle portion of the spike, the ranges were 20.6 to 51.7°, 30.9 to 38.7°, and 26.3–54.9°, respectively; in the upper‐middle portion, the ranges were 17.3 to 51.8°, 30.6 to 41.0, and 19.1 to 50.5°, respectively; and in the top portion of the spike, the ranges were 8.0 to 44.75°, 23.3 to 30.8°, and 15.1 to 71.5°, respectively (Figure S3a, Supporting Information). These results suggest that basal spikelets tend to remain closer to the main stem of the spike, but the upper spikelets form wider angles to better capture sunlight. This characteristic has significant potential for application in agricultural production.^[^ ^23^ ^]^

The gap between spikelets on the rachis is thought to be related to grain size.^[^ ^24^ ^]^ Using a method similar to that used to measure spikelet angle above, we classified the spikelet gap of each line in wild‐type plants and the EMS population (Figure 2e, Figure S2e, Supporting Information). In wild‐type plants, the spikelet gap at the bottom, lower‐middle, upper‐middle, and top positions were 0.40 to 0.60, 0.38 to 0.49, 0.36 to 0.50, and 0.23 to 0.40 mm, respectively. In the EMS population, the spikelet gap at the equivalent positions was 0.25 to 0.71, 0.32 to 0.59, 0.24 to 0.64, and 0.13 to 0.95 mm, respectively. The distribution of spikelet gap types was relatively even, with three main types identified, namely DNDD, DIDI, and DNDI. These three spikelet gap types accounted for 20.1%, 19.9%, and 19.7% of all lines in the EMS population, respectively. The ranges of spikelet gaps for the three types, DNDD, DIDI, and DNDI, were as follows: in the bottom position, the gaps ranged from 0.38 mm to 0.71 mm, 0.34 mm to 0.62 mm, and 0.39 mm to 0.66 mm; in the lower‐middle position, 0.35 mm to 0.59 mm, 0.33 mm to 0.52 mm, and 0.32 mm to 0.53 mm; in the upper‐middle position, 0.31 mm to 0.60 mm, 0.24 mm to 0.52 mm, and 0.24 mm to 0.48 mm; and in the top position, 0.14 mm to 0.48 mm, 0.26 mm to 0.92 mm, and 0.13 mm to 0.86 mm, respectively (Figure S3b, Supporting Information). An analysis of the correlation between different phenotypes revealed a significant relationship between spikelet angle and TKW, as well as between spikelet gap and GN. Indeed, we obtained a significant difference from a comparison of TKW values across subgroups with different spikelet angles (*P*‐value = 0.032). The average TKW of wild‐type plants was 44.61 g, whereas the average TKW of spikelet angle subtypes ranged from 42.10 g to 43.17 g (Figure 2f). Similarly, we observed notable differences (*P*‐value = 0.047) in GN among subgroups with varying spikelet gaps. The average GN of wild‐type plants was 27.86, compared to 25.52 to 26.42 for the spikelet gap subtypes (Figure 2g). These findings highlight the potential links between geometric traits and agronomic traits, offering new insights into the mechanisms underlying the formation of yield‐related traits.

### 2.4 Creating an Updated Genotyping Dataset Based on Optimized Filters for Association Studies

Our previous work aimed to establish a reliable mutant germplasm resource by applying stringent filtering criteria during genotyping, retaining only mutation sites unique to individual lines. Although this approach improved the accuracy of EMS‐induced mutation identification, it inevitably sacrificed many mutation sites with probabilities above 90% but below 100%.^[^ ^20^ ^]^ To use the KN9204 EMS population for association mapping, we reassessed all mutations in the lines using exome‐capture sequencing data generated in our previous work, with the goal of calculating a more reasonable filtering threshold that mitigates the loss of mutation information due to our previous very strict filtering criteria.^[^ ^20^, ^25^ ^]^ We used the original genotype data for simulation to predict that a mutation site could simultaneously occur in several lines. Through a permutation test (Figure S4a, Supporting Information), we determined that identifying the same mutation site in fewer than 0.7% of the total population of lines is highly likely (99% confidence interval). Therefore, we adopted this new filtering criterion to assemble our genotyping dataset.

**Figure 3:**
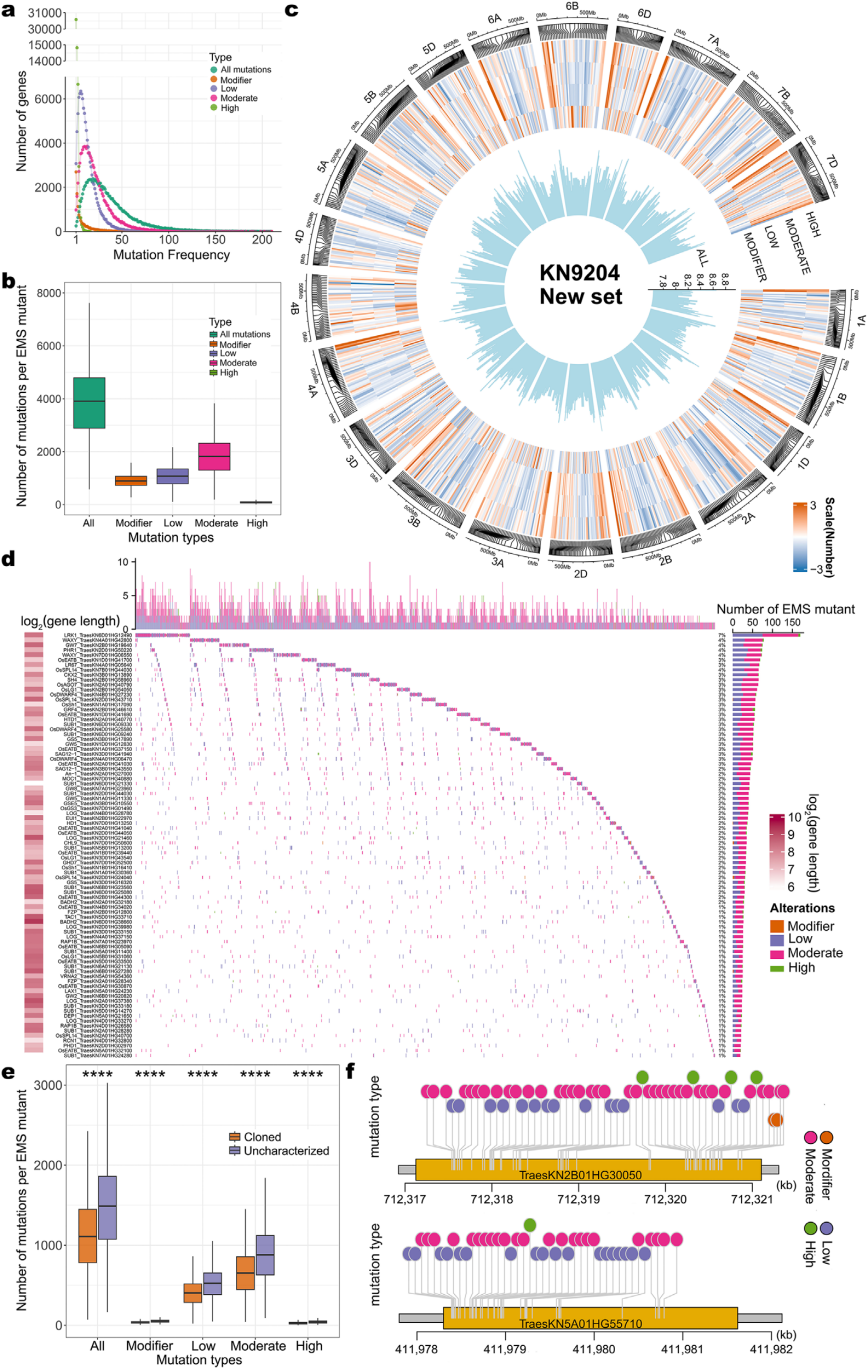
Construction of a new genotyping dataset for association analysis. a) The frequency of different mutation effects in each gene, the mutation effects are predicted using SnpEff. b) Frequency of different mutation effect types in each EMS mutant. c) Circos plots showing the density of mutation effect types across the genome. From the innermost to outermost circles: the logarithm value of total number of mutation sites, normalized values for Modifier, Low, Moderate, and High mutation effect sites, and density within a 100‐gene window. d) Mutation patterns of genes homologous to functional genes across different genotypes, with gene length on the left and total mutation count in the population on the right, the top part represents the number of functional gene mutations present in a single sample. e) Number of mutations in genes homologous to functionally characterized or uncharacterized genes in the population based on mutation types. f) Examples of mutation patterns in two candidate genes associated with TKW in the KN9204 EMS population. Each box plot represents the median (center line), interquartile range (box), and minimum/maximum values excluding outliers (whiskers). Outliers are shown as individual dots.

With this new filtering criterion, we discovered 4 228 167 mutation sites in 1839 lines, with 280 747 different sites detected in at least five lines (Figure S4b, Supporting Information). The percentage of EMS‐type mutation sites (G/A or C/T transitions) was 88.9% (Figure S4c, Supporting Information). For comparison, our previous dataset identified 1 605 107 mutation sites, with no site detected in more than one line. This substantial increase in genotyping data depth highlights the critical role of SNP calling and filtering in population genomics studies. The most frequent number of mutation sites per gene was 19, observed for 2389 genes (**Figure** **3**a). Using SNPeff,^[^ ^26^ ^]^ we classified the mutation sites into four mutation types depending on their functional consequences (low, moderate, high, and modifier). We also categorized the mutation sites based on gene annotation (Figure S4d, Supporting Information). On average, each gene contained 36.6 mutations, with the majority classified as having moderate effects (accounting for 59.7% of all mutation sites) (Figure S4e, Supporting Information). Each line had 4072 mutations on average, including an average of 1903 moderate‐effect mutations and 86 high‐effect mutations per line (Figure 3b). The highest number of mutation sites identified in a single line was 29 384 (Figure S4f, Supporting Information). The density of mutation sites was lower from the ends of chromosomes toward the centromere regions (Figure 3c). This updated genotyping dataset resulted in a significantly higher detection of mutation sites across the population, thereby facilitating more effective gene–phenotype association analysis.

### 2.5 Determining Gene Function by Comparative Analysis of Protein Sequences

We hypothesized that genes in KN9204 that are homologous to cloned genes in other species are more likely to influence agronomic traits than genes of unknown function. We identified homologs of previously cloned genes through BLAST searches with the protein sequence; we refer to these genes as “functional genes” in this study. Among the 110 326 high‐confidence genes in the KN9204 genome, 39 752 genes are considered to be functionally characterized. In the EMS mutant population, we detected mutations in these genes in multiple lines (Figure 3d), thus allowing us to test for a correlation between mutation frequency and phenotypic variation. On average, each gene was mutated in about 39 lines (ranging from 19 to 135). In individual lines, each gene had an average of 2 mutations (ranging from 0 to 10). Additionally, there was no clear relationship between the number of mutations covered by similar homologs and gene length, suggesting that EMS mutation might be affected by biological factors (Figure 3d).

We randomly selected two groups of genes: a subset of the functional genes, and an equal number of genes in the KN9204 genome with no homology to cloned genes. We performed this step 100 times and examined the differences in the number of mutation sites per gene from the two groups each time. Using *t*‐tests, we detected significant differences between these two groups, including for the total number of mutations per gene and the number of mutations for each mutation type (Figure 3e). Indeed, the average number of mutations in 39 752 functional genes across the entire population was 1171, which was significantly lower than the 1572 mutations for genes of unknown function (*P*‐value < 0.01). We speculate that functional genes play more important regulatory roles in wheat growth and development than randomly selected genes (Figure 3f). Moreover, an excess of mutations could lead to a loss of gene function, thereby affecting plant survival. As a result, lines with many mutations in functional genes may not have survived during the creation of the mutant library.

### 2.6 Association Between Mutations in Genes and Phenotypes Identifies Abundant Candidate Genes

**Figure 4:**
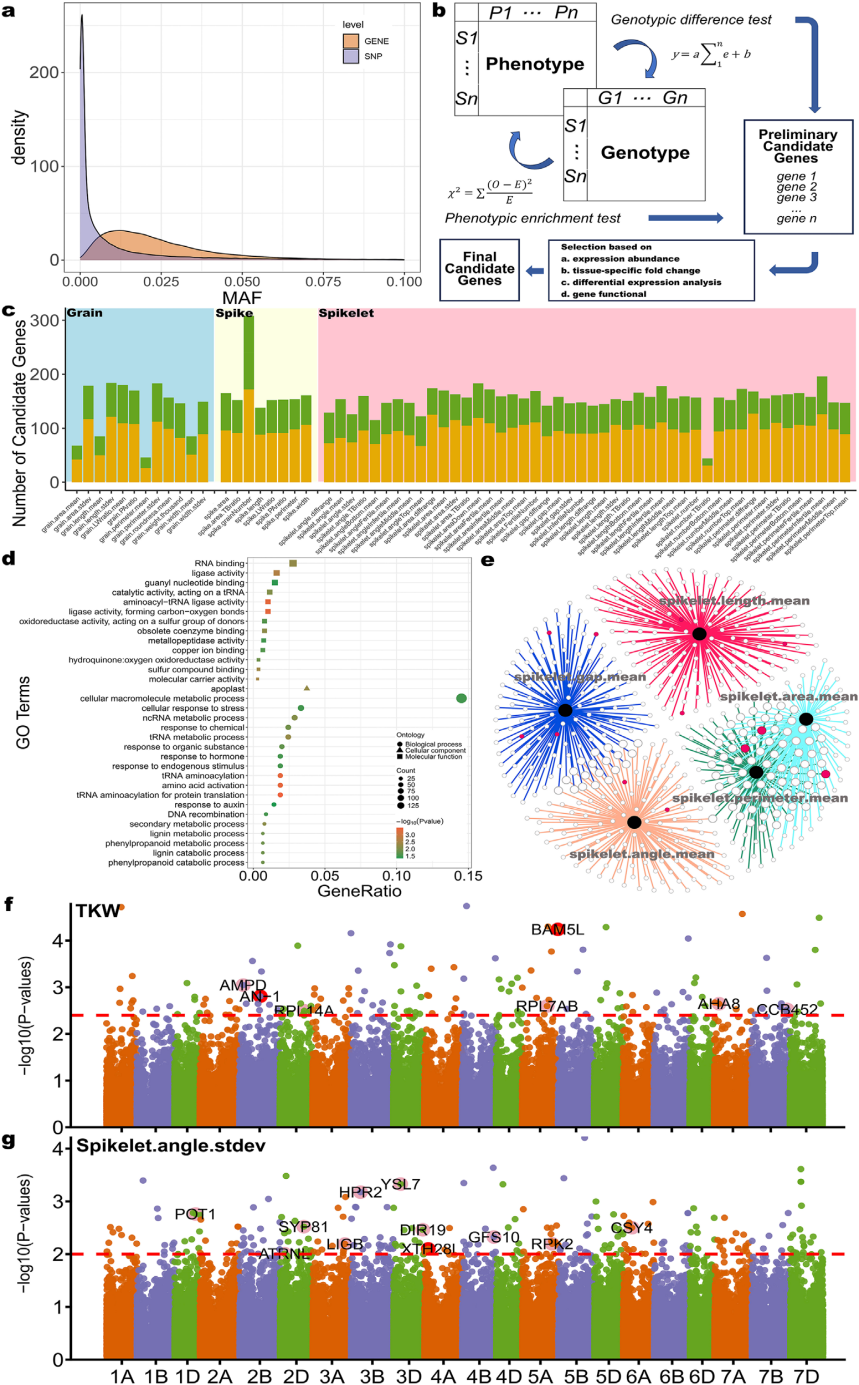
Methods of gene‐level association analysis. a) Distribution of MAF at the single‐nucleotide polymorphism (SNP) level and the gene level in the KN9204 EMS population. Gene‐level aggregation alleviates the problem of extremely low MAF for individual SNPs. b) Diagram illustrating the GH‐GLA association analysis framework. A linear model is used to test the genotypic effect of each gene on phenotypes, which is complemented by a phenotypic enrichment test. The preliminary candidate genes obtained can be further selected using transcriptome data based on tissue‐specific expression patterns or functional annotations of homologous genes to yield the final results. c) Number of candidate genes for each phenotype from the association analysis. Traits are grouped by phenotypic categories: grain (blue), spike (yellow), and spikelet (pink), with genes homologous to functionally characterized genes (yellow) or uncharacterized genes (green) shown. d, e) Manhattan plots showing the association results for TKW (d) and the standard deviation of spikelet angle (e). The dashed red lines indicate the significance threshold, which was determined by permutation test. f) Gene ontology (GO) term enrichment analysis results of candidate genes related to spikelet‐associated traits. Dot size represents the number of genes, and color indicates statistical significance. g) Association network of candidate genes for spikelet geometric phenotypes. Each node represents a candidate gene. Red nodes indicate candidate genes homologous to a functionally characterized gene, and the size of each node represents the number of phenotypes each gene is associated with.

GWAS is widely employed to identify SNPs associated with complex genetic traits and diseases.^[^ ^27^ ^]^ For GWAS, loci with a MAF greater than 5% are generally considered suitable for statistical analysis. Although our population comprises 1839 lines and EMS mutagenesis offers advantages such as high efficiency, high mutation frequency, and broad mutational spectrum, the likelihood of generating loci with high MAF remains low due to the large genome size of hexaploid wheat. In this mutant population, 95% of all loci have a MAF below 0.01 (**Figure** **4**a), which presents substantial limitations for traditional GWAS. Mutations at different positions within the same gene can affect its function and consequently alter phenotypes. The average mutation frequency in genes was 2.6%, which was higher than the MAF of individual mutation sites (average 0.98%), making it feasible to perform gene–phenotype association analysis. Therefore, we established a gene‐level gene–phenotype association analysis framework, named Gene Hunter based on Gene‐Level Association (GH‐GLA) (Figure 4b, Figure S5a,b, Supporting Information). Conventional approaches typically rely on manually identifying individuals with phenotypic mutations and detecting shared mutations among them. Compared to GH‐GLA, these methods require exceptionally strong phenotypic effects and high penetrance to enable gene mapping. As a result, the number of candidate genes identified is often limited (Figure S5c, Table S4, Supporting Information). In the GH‐GLA framework, candidate genes obtained from both pathways are cross‐validated to preliminarily identify potential regulators of the phenotype. We subsequently leveraged publicly available transcriptome datasets from diverse tissues to select candidate genes based on tissue‐specific expression patterns and functional annotations of homologous genes, thereby prioritizing targets with higher biological plausibility.^[^ ^25^ ^]^ We have encapsulated GH‐GLA into an R package (https://github.com/gaze‐abyss/GH‐GLA) to facilitate the analysis of our large‐scale data.

We conducted an association analysis for all phenotypes across the entire EMS population to identify their respective candidate genes (Figure 4c, Figure S6a, Table S5, Supporting Information). GH‐GLA yielded 175 candidate genes for TKW, 141 for GN, 143 for spikelet number, 129 for spike perimeter, and 73 for grain area. Notably, many candidate genes have homologs in other plant species with established roles in modulating yield‐related traits. Representative examples include Protein phosphatase 2CA (PP2CA) involved in abscisic acid (ABA) signaling,^[^ ^28^ ^]^ the RNA interference mediator Argonaute 1 (AGO1),^[^ ^29^ ^]^ and the sugar transporter Sugars will eventually be exported 17 (SWEET17),^[^ ^30^ ^]^ suggesting that these i‐traits may underlie fundamental components of yield architecture. We discovered TraesKN2B01HG30050 and TraesKN5A01HG55710 as two candidate genes associated with TKW through GH‐GLA (Figure 3f). Protein sequence alignment indicated that the proteins encoded by TraesKN2B01HG30050 and TraesKN5A01HG55710 are highly similar to *Awn‐1* (*AN‐1*)^[^ ^31^ ^]^ in rice and beta‐amylase 5 (BAM5),^[^ ^32^ ^]^ respectively (Figure 4d). Therefore, we named these genes *TaAN‐1* and *TaBAM5L* (for *BAM5‐like*), respectively. In the mutant library, many of the mutation sites were located in these two genes.

For the standard deviation of spikelet angle, the candidate genes included *YSL7* (TraesKN3D01HG15700)^[^ ^33^ ^]^ and *XTH28L* (TraesKN4A01HG06820)^[^ ^34^ ^]^ (Figure 4e). For GN and the mean spikelet gap traits, the candidate genes identified included *FPF1* (TraesKN3A01HG46780),^[^ ^35^ ^]^ *WRKY6* (TraesKN3D01HG16350),^[^ ^36^ ^]^ *UBA2* (TraesKN4D01HG13710),^[^ ^37^ ^]^ and the sucrose transporter gene *SUC3* (TraesKN1D01HG14320)^[^ ^38^ ^]^ (Figure S6b,c, Supporting Information), demonstrating the reliability and effectiveness of our pipeline for candidate gene identification.

We performed a GO term enrichment analysis on all candidate genes (Figure S6d, Supporting Information). For the candidate genes related to spikelet traits, the enriched GO terms across the biological process, cellular component, and molecular function categories included “RNA binding,” “apoplast,” and “ncRNA metabolic process” (Figure 4f). For the candidate genes related to grain traits and spike traits, the enriched GO terms were mainly concentrated in the cellular component categories “extracellular region” and “intracellular anatomical structure” (Figure S6e,f, Supporting Information).

We then compared the phenotypes of lines carrying mutations in these candidate genes to those of other EMS lines with no mutations in these genes, which revealed statistically significant phenotypic differences that validate the reliability of our candidate gene identification (Figure S7, Supporting Information).

We observed a substantial overlap among the candidate genes identified for the two spike geometric traits of spikelet perimeter and spikelet area. There was also some overlap among the candidate genes for spikelet angle and spikelet gap. This finding suggests that some genetic factors may simultaneously influence multiple related traits, whereas others may be more specific to certain geometric characteristics of the spike (Figure 4g).

### 2.7 Validation of Candidate Genes for Thousand‐kernel Weight and Spikelet Angle

**Figure 5:**
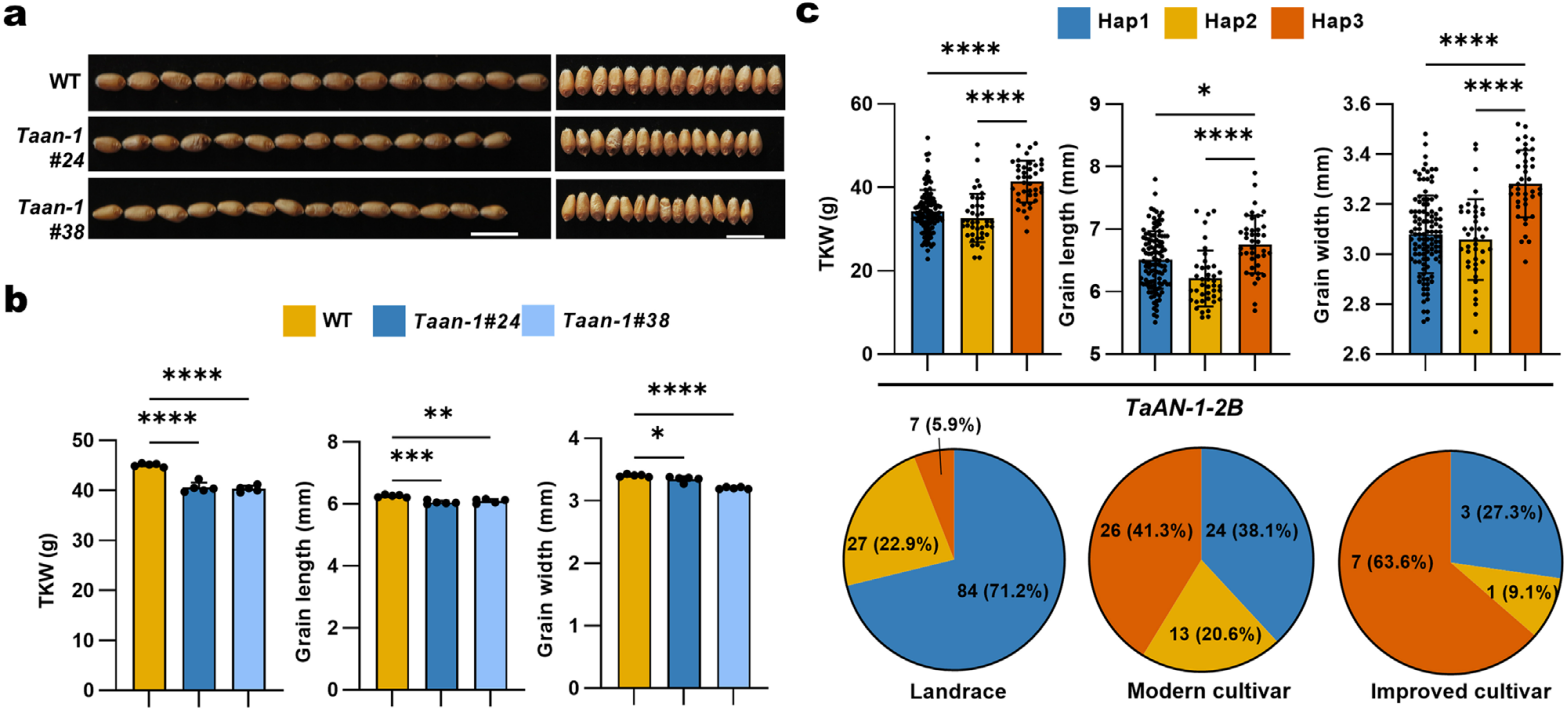
The candidate gene TaAN‐1 affects thousand‐kernel weight in wheat. a) Representative photographs showing GL and GW for the wild type (KN9204) and two *Taan‐1* (*aabbdd*) mutant lines. Scale bars, 1 cm. b) TKW, GL, and GW of KN9204 and *Taan‐1* (*aabbdd*) mutant lines. Data are means ± standard deviation (SD). At least five plants were performed (****, *p* < 0.0001; ***, *p* < 0.001; **, *p* < 0.01; *, *p* < 0.05, as determined by one‐way ANOVA). c) Top, TKW, GL, and GW values as a function of the *TaAN‐1‐2B* haplotypes within 262 accessions from the Chinese wheat mini‐core population. Bottom, pie charts showing the number and percentage of the three haplotypes. Data are means ± SD. ****, *p* < 0.0001; *, *p* < 0.05; ns, not significant, as determined by one‐way ANOVA). Each dot represents an individual plant or accession.

We investigated the function of the TKW‐associated gene TraesKN2B01HG30050 located on chromosome 2B. The protein encoded by TraesKN2B01HG30050 is highly similar to the basic helix‐loop‐helix (bHLH)‐type transcription factor AN‐1 in rice, which regulates awn development, grain size, and grain number.^[^ ^39^ ^]^ Transcriptome deep sequencing (RNA‐seq) data indicated that *TaAN‐1* is expressed in roots, stems, and spikes (Figure S8a, Supporting Information).^[^ ^25^ ^]^ We used the CRISPR/Cas9 system to generate knockout mutants in all three homoeologs of *TaAN‐1* in the KN9204 background (Figure S9a, Supporting Information). Sanger sequencing identified two independent mutant lines carrying frameshift mutations in all three *TaAN‐1* homoeologs. The resulting *Taan‐1* edited lines exhibited significantly lower values for TKW, GL, and GW (**Figure** **5**a,b). This phenotype mirrors that of the rice *an‐1* mutant, confirming the reliability of our screening approach.

Phylogenetic analysis revealed that proteins related to TaAN‐1 from dicot and monocot species form distinct clusters (Figure S8b, Table S6, Supporting Information), suggesting potential functional divergence between dicot‐ and monocot‐derived TaAN‐1 members. To elucidate the role of *TaAN‐1* in breeding programs, we performed a haplotype analysis followed by association analysis of agricultural traits. We examined the haplotypes of *TaAN‐1‐2B* in the wheat genome resequencing data from Li,^[^ ^40^ ^]^ representing a mini‐core collection of 262 wheat accessions. We detected three main haplotypes (Hap1, Hap2, and Hap3) for *TaAN1‐2B* (Figure S9d, Supporting Information). We then conducted an association analysis between the haplotypes at *TaAN1‐2B* and several agronomic traits in the same mini‐core collection.^[^ ^40^ ^]^ Accessions harboring Hap3 showed significantly higher values for TKW, GL, and GW, suggesting *TaAN1‐2B* Hap3 as a favorable haplotype for TKW and seed size (Figure 5c, top). To further investigate the deployment of *TaAN1‐2B* haplotypes during breeding in China, we evaluated the frequency of *TaAN1‐2B* haplotypes using the 262‐accession mini‐core collection. The frequency of *TaAN1‐2B* Hap3 rose from landraces to modern cultivars (Figure 5c, bottom). This result suggests that TaAN1‐2B Hap3 underwent selection during breeding to increase TKW and seed size.

**Figure 6:**
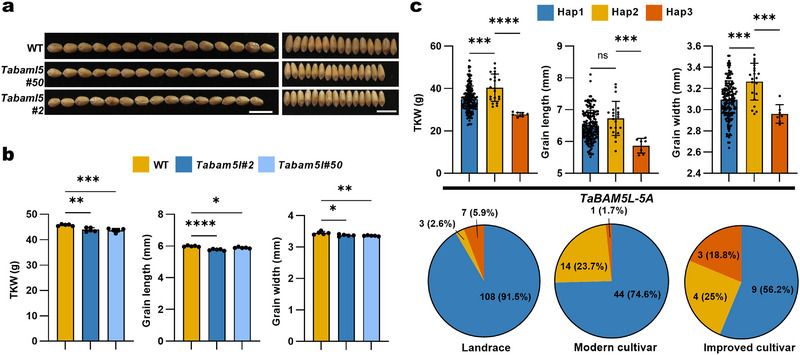
The candidate gene *TaBAM5‐like* affects thousand‐kernel weight in wheat. a) Representative photographs showing GL and GW of wild type (KN9204) and *Tabam5l* (*aabb*) mutant lines. Scale bars, 1 cm. b) TKW, GL, and GW of KN9204 and *Tabam5l* (*aabb*) mutant lines. Data are means ± SD. At least five plants were assessed (****, *p* < 0.0001; ***, *p* < 0.001, **p < 0.01, *p < 0.05, as determined by one‐way ANOVA). c) TKW, GL, and GW values as a function of the *TaBAM5L‐5A* haplotypes within 262 accessions from the Chinese wheat mini‐core population (top). Pie charts show the number and percentage of the three haplotypes (bottom). Data are means ± SD (****, *p* < 0.0001; ***, *p* < 0.001; ns, not significant, as determined by one‐way ANOVA). Each point represents an individual plant or accession.

We then turned to another TKW‐associated gene, TraesKN5A01HG55710, located on chromosome 5A, as it exhibited the highest expression level in seed tissues among all candidate genes. Protein sequence alignment confirmed that TraesKN5A01HG55710 encodes a β‐amylase that is 62% identical to the characterized BAM5 from Arabidopsis.^[^ ^41^ ^]^ Arabidopsis BAM5 plays a significant role in leaf starch metabolism and plant growth.^[^ ^42^ ^]^ However, whether BAM5 possesses amylase activity and whether its homologs in crops have specific functions remain unknown. We named TraesKN5A01HG55710 as *TaBAM5‐like* (*TaBAM5L*). RNA‐seq data indicated that *TaBAM5L* is specifically and highly expressed in seeds at 14, 21, and 28 days after anthesis (DAA) (Figure S8a, Supporting Information).^[^ ^25^ ^]^ We obtained knockout lines with frameshift mutations in two of the homoeologs of *TaBAM5L* in the KN9204 background using the CRISPR/Cas9 system (Figure S9b, Supporting Information), as confirmed by Sanger sequencing. The edited *Tabam5l* lines displayed significantly lower values for TKW, GL, and GW (**Figure** **6**a,b). As with TaAN‐1, phylogenetic analysis revealed that proteins related to TaBAM5L from dicot and monocot species form distinct clusters (Figure S8c, Table S7, Supporting Information), suggesting potential functional divergence between BAM5L members from dicots and monocots. To investigate the role of *TaBAM5L* in breeding improvement, we performed a haplotype analysis using the 262‐accession mini‐core population, identifying three major haplotypes for *TaBAM5L* (Figure S9e, Supporting Information).^[^ ^40^ ^]^ Association analysis showed that *TaBAM5L‐5A* Hap2 is associated with significantly higher values for TKW, GL, and GW (Figure 6c, top). However, *TaBAM5L‐5A* Hap2 only accounted for 10.9% of the total population, indicating that this favorable haplotype is currently underutilized in breeding programs and has high potential for further molecular‐assisted breeding to raise TKW and crop yield (Figure 6c, bottom).

**Figure 7:**
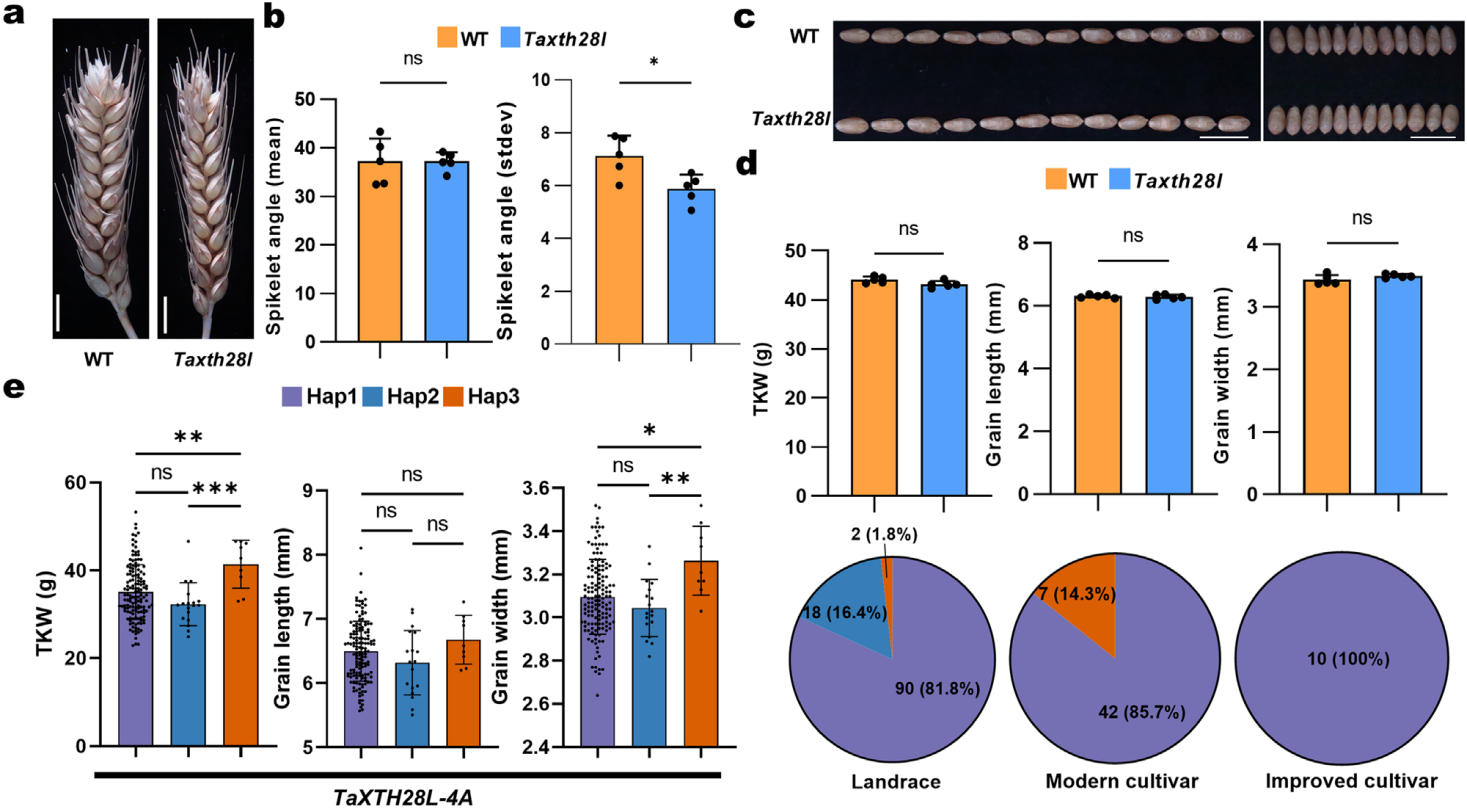
The candidate gene *TaXTH28‐like* affects the spikelet angle of wheat. a) Representative photographs of spikes from wild type (KN9204) and *Taxth28l* (*aabbdd*) mutant lines. Scale bars, 1 cm. b) Spikelet angle and standard deviation of the spikelet angle of KN9204 and *Taxth28l* (*aabbdd*) mutant lines. Data are means ± SD. At least five independent biological replicates were performed (*, *p* < 0.05; ns, not significant, as determined by one‐way ANOVA). c) Representative photographs of GL and GW of wild type (KN9204) and *Taxth28l* (*aabbdd*) mutant lines. Scale bars, 1 cm. d) TKW, GL, and GW of KN9204 and *Taxth28l* (*aabbdd*) mutant lines. Data are means ± SD. At least five plants were assessed (ns, not significant, as determined by one‐way ANOVA). e) TKW, GL, and GW values as a function of *TaXTH28L‐4A* haplotypes within 262 accessions from the Chinese wheat mini‐core population (left). Pie charts show the number and percentage of the three haplotypes (right). Data are means ± SD. ***, *p* < 0.001; **, *p* < 0.01; *, *p* < 0.05; ns, not significant, as determined by one‐way ANOVA). Each point represents an individual plant or accession.

Finally, we selected the spikelet angle‐associated gene TraesKN4A01HG06820. This gene encodes the xyloglucan endotransglucosylase/hydrolase XTH28, with no prior functional characterization reported in wheat. We designated this gene as *TaXTH28‐like* (*TaXTH28L*). RNA‐seq data demonstrated that *TaXTH28L* is specifically and highly expressed in spikes at the booting stage, spikes at the shooting stage, and seeds at 7 and 14 DAA (Figure S8a, Supporting Information).^[^ ^25^ ^]^ Using CRISPR/Cas9, we generated deletion mutations in all three *TaXTH28L* homoeologs (TraesKN4A01HG06820; TraesKN4B01HG26850; TraesKN4D01HG25120) in the KN9204 background (Figure S9c, Supporting Information). The edited lines showed no significant difference in spikelet angle, but the spikelet angle values had a significantly smaller standard deviation, indicating a looser spikelet arrangement in the mutants (**Figure** **7**a,b). Further examination of grain traits in the *Taxth28l* mutant lines showed no significant differences from the wild type in terms of TKW, GL, or GW values (Figure 7c,d). A phylogenetic analysis indicated that proteins related to TaXTH28L from dicots and monocots form distinct clades, suggesting potential functional divergence between these groups (Figure S8d, Table S7, Supporting Information). To assess the breeding value of *TaXTH28L*, we performed a haplotype analysis using the 262‐accession mini‐core population, identifying three main haplotypes in *TaXTH28L‐4A* (Figure S9f, Supporting Information). Hap3 of *TaXTH28L‐4A* was associated with significantly higher values for TKW and grain width (Figure 7e, left). However, despite being an elite haplotype, Hap3 showed no significant selective sweep in landraces, modern cultivars, or improved cultivars (Figure 7e, right).

### 2.8 Detection of Epistasis Between Gene Pairs Using the EMS Panel Reveals Network Modules Involved in Yield Component Traits

**Figure 8:**
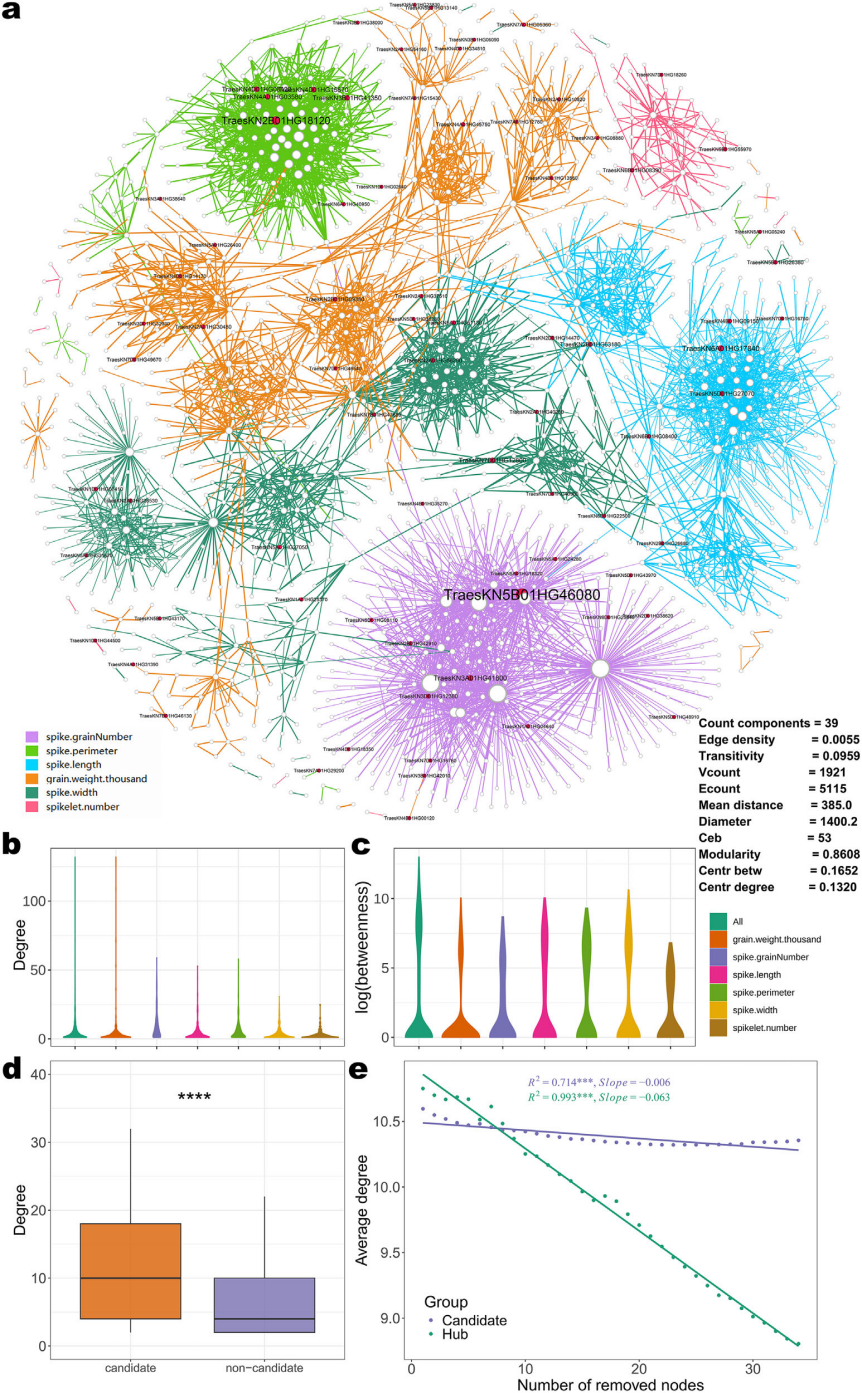
Construction of gene‐level epistatic interaction networks. a) Construction of an epistatic interaction network at the gene level. The color of the edges represents the phenotype, and the thickness of the edges indicates the significance of the epistatic gene pairs, with thicker edges representing more significant interactions. The node size reflects the number of gene pairs, and red nodes represent candidate genes identified through GH‐GLA. Network topology metrics are listed in the bottom right corner. b) Violin plots showing the distribution of nodes grouped by six major trait categories, revealing hub‐like gene nodes within certain phenotypic modules. c) Violin plots showing the betweenness centrality levels of gene nodes in different phenotype modules, the top part highlighting the presence of bottleneck nodes with trait‐specific centrality patterns. d) Degree centrality of GH‐GLA candidate genes and non‐candidate genes. Candidate genes tend to have significantly more degrees than non‐candidate genes (****, *p* < 0.0001). Each box plot represents the median (center line), interquartile range (box), and minimum/maximum values excluding outliers (whiskers). e) Assessment of network analysis by iterative node removal. The average degree declines more rapidly when hub nodes are removed than when candidate gene nodes are removed, indicating that hubs play a critical role in maintaining network connectivity.

Epistasis reflects non‐linear genetic interactions between genes and provides critical insights into the architecture of complex traits. We therefore constructed an epistatic interaction network for the EMS mutant population based on individual phenotypes (**Figure** **8**a, Table S8, Supporting Information). Phenotypes under strong genetic control often form larger epistatic interaction networks, and the genes within these phenotypic networks also have a high node degree, such as TKW (Figure 8b). In the epistatic interaction network for spike grain number, many genes showed a higher betweenness than those in the spike number network and formed a network module (Figure 8c, Table S9, Supporting Information). GO term enrichment analysis detected over‐represented functional terms among these network modules, including “protein phosphorylation” and “metal ion transmembrane transporter activity” (Table S10, Supporting Information). The genes marked in red in the network are candidate genes identified through GH‐GLA (Figure 8a). These candidate genes rarely appeared in the central positions of the network, suggesting that they are not hub nodes, although the degree of these candidate genes was significantly higher (*P*‐value < 0.01) than that of an equally sized set of randomly selected (non‐candidate) genes (Figure 8d). We analyzed the consequences of deleting two types of genes from the network. We conducted a simulation, which showed that deletion of hub genes led to a significantly faster drop in the network average degree compared to the deletion of candidate genes (Figure 8e). By mining information from the epistatic interaction network, we can better understand the genetic basis and gene interaction mechanisms of organisms. These data provide more insights into the molecular basis of phenotype formation and offer more options for molecular breeding.

## 3 Discussion

### 3.1 Phenomics Facilitates the Precise Identification of Phenotypes

Wheat varieties globally were largely shaped by long‐term selection from breeders, which resulted in loss of genetic diversity due to bias in artificial selection.^[^ ^43^ ^]^ EMS mutagenesis addresses this limitation by creating diverse genotypes and phenotypes, expanding genetic diversity and facilitating the identification of beneficial traits that may enhance crop performance and adaptability.^[^ ^44^ ^]^ Despite this diversity, we observed limited variation in spike morphology, suggesting constraints on the architecture of plant organs. These architectural limitations may serve as a blueprint for breeding strategies. Understanding these limitations can guide breeders in selecting feasible phenotypes and improve the efficiency of breeding programs. In the past, the absence of image‐based phenotyping technologies hindered the accurate classification of spike morphology.^[^ ^19^, ^45^ ^]^ In this study, we detected different spike types across the EMS population based on the distribution of spikelet angle and spikelet gap, for which precise values are difficult to obtain without using automated phenomics‐based methods. However, even such minor geometric variations may contribute to the phenotypic diversity observed across different lines.

### 3.2 GH‐GLA Enables High‐Precision Candidate Gene Identification within the EMS Population

Traditional forward genetics methods, such as GWAS and quantitative trait locus (QTL) mapping, have inherent limitations when exploring genotype–phenotype relationships. GWAS can be biased by population structure, leading to false positives,^[^ ^46^ ^]^ whereas QTL mapping is restricted to alleles within typically two parental lines.^[^ ^47^ ^]^ EMS populations, with their high genetic diversity and lack of genetic structure, overcome these issues. However, the random nature of EMS‐induced mutations results in a low MAF for mutations, complicating direct association analysis. We propose shifting the focus from SNPs to gene‐level analysis, which is more effective in EMS populations with higher gene‐level MAF. We performed molecular experiments to validate the function of three candidate genes. We developed GH‐GLA as a generalized tool for analyzing EMS‐mutagenized populations across diverse crop species. The pipeline requires only standard input formats (genotype VCF and matched phenotypic data), making it readily transferable to EMS populations in virtually any crop species. We therefore anticipate that GH‐GLA can be effectively applied to accelerate gene discovery and functional characterization in diverse plant systems. Furthermore, our results demonstrate that integrating phenomics data enables high‐precision candidate gene identification. For instance, analysis based solely on spike length and width identified only 251 genes, whereas incorporating a comprehensive set of spike‐related traits elevated this number to 1416, significantly expanding genetic resources (Table S5, Supporting Information). The investigation of mutations in non‐coding DNA and their effects on phenotype holds great promise for elucidating gene regulatory mechanisms and deciphering the genetic basis of hereditary diseases and complex traits. Although these regions do not encode proteins, they serve as critical regulatory hubs governing gene expression, and their mutation can disrupt transcriptional networks by modulating transcription factor binding, chromatin state, and non‐coding RNA functions. Indeed, GH‐GLA should be effective as a highly extensible computational framework that can readily incorporate association analysis modules for non‐coding regions, enabling users to evaluate relationships between non‐coding variants and phenotypes. However, this expanded functionality also imposes more stringent requirements on the quality of genotyping data and annotation completeness, particularly regarding the annotation of regulatory elements (enhancers/silencers), the accuracy of variant calling in repetitive regions, and haplotype phasing for *cis*‐regulatory analysis.

### 3.3 Using the Epistatic Network to Investigate the Genetic Basis of Phenotypes

Understanding complex traits involves interpreting gene interactions, or epistasis.^[^ ^48^ ^]^ Traditional methods often fall short in capturing the complexity of these interactions.^[^ ^49^ ^]^ EMS populations, with their random mutations, allow the exploration of epistatic interactions, which are non‐additive interactions between genes that nevertheless affect phenotype. Unlike biparental populations, EMS populations provide broader allelic diversity, enabling the identification of new genetic interactions and networks. Despite the challenge of calculating epistatic interactions across numerous SNPs, focusing on gene pairs is feasible and reveals the existence of network modules. This approach identifies complex, non‐linear regulatory patterns and synergistic effects among traits. Integrating epistatic network modules across traits reveals a larger biological network, highlighting gene pleiotropy and synergies among phenotypes. However, it should be noted that the synergistic relationships between genes reported here were derived solely from computational methods, and the phenotypic effects of such interactions remain to be biologically tested. Targeted experiments should be designed to better elucidate the underlying genetic mechanisms.

### 3.4 Synergistic Effects of *TaAN‐1*, *TaBAM5L*, and *TaXTH28L* on Wheat Yield Architecture Traits

Our functional validation of *TaAN‐1*, *TaBAM5L*, and *TaXTH28L* confirms the efficacy of mutant library screening for identifying yield‐associated genes and reveals distinct pathways for wheat improvement. *TaAN‐1* (encoding a bHLH transcription factor) and *TaBAM5L* (encoding a β‐amylase) likely regulate TKW through conserved mechanisms controlling grain dimensions, and the characterization of *TaXTH28L* uncovered a previously unknown dimension of yield architecture: spatial spikelet arrangement. Mutation of *TaXTH28L* significantly changed the variation in spikelet angle without compromising TKW or grain size, demonstrating that spikelet packing density can be genetically decoupled from grain yield. This phenotype holds substantial breeding relevance: optimizing spikelet arrangement through *TaXTH28L* engineering may enhance disease resilience by improving the spike microclimate (e.g., lowering humidity for Fusarium resistance) and boost harvest efficiency through uniform panicle morphology. The elite haplotypes identified—*TaAN1‐2B* Hap3 (increasingly selected in modern cultivars), *TaBAM5L‐5A* Hap2 (underutilized), and *TaXTH28L‐4A* Hap3 (associated with higher TKW and grain width)—collectively offer the potential for synergistic improvement of multiple traits. Notably, the lack of historical selection for *TaXTH28L‐4A* Hap3 highlights untapped value in targeting this spike architecture gene for future ideotype breeding.

This study combines EMS mutagenesis with deep learning–driven phenotyping to explore the range of wheat agronomic traits and the genetics behind variation in phenotypes. We developed the GH‐GLA framework for gene‐level association analysis, overcoming challenges in EMS populations and identifying candidate genes for wheat traits. To the best of our knowledge, this is the first association mapping work based on an EMS library in wheat, and this method can also be applied to other crops.

## 4 Experimental Section

### Plant Growth Conditions and Phenotyping Methods

To obtain field phenotypic data, a total of 1839 KN9204 mutant lines and 15 plants from the parental line KN9204 were sown during the 2022–2023 growing season in Zhao County, Hebei Province, China (38°04′N, 114°28′E). Each mutant line was planted in a 1.5‐meter single‐row plot with a plant spacing of 10 cm and a row spacing of 25 cm. For each line, the main spike (primary tiller) was excised from five plants after harvest and photographed under standardized conditions. For each plant, one front‐view image and one side‐view image were taken, resulting in 20 963 raw images consisting of 18 388 spike images and 2575 whole‐plant images. After manual quality control to remove substandard images, 9145 front‐view and 9146 side‐view spike images along with 2084 whole‐plant images were retained. These curated images were processed using a deep learning–based phenomics pipeline to extract the phenotypic data used in this study.

The *Taan‐1*, *Tabam5l*, and *Taxth28l* mutants were generated in the KN9204 background. Single guide RNA (sgRNA) sequences were selected based on the GenBank sequences for each target gene, and used to conduct CRISPR/Cas9‐based genome editing in wheat. The chosen sgRNAs were also used as queries for a BLAST search against the three wheat subgenomes to determine whether the sgRNAs would target homoeologs in the three wheat sub‐genomes. The target sites were amplified by PCR and sequenced via Sanger sequencing to confirm the mutations (Table S11, Supporting Information).

Plants from the wild type and the *Taan‐1*, *Tabam5l*, and *Taxth28l* mutants were grown in a greenhouse under long‐day conditions (16‐h light/8‐h dark photoperiod) with about 60% relative humidity and a light intensity of ≈200 µmol m^−2^ s^−1^ after full vernalization. The growth temperature was set to 18 °C (day)/12 °C (night) before the stem elongation stage and 22 °C (day)/16 °C (night) after the stem elongation stage. At the grain‐filling stage, the temperature cycle was 26 °C (day)/18 °C (night).

### Image‐Based Phenotyping

In this study, image analysis techniques were used to extract traits related to plant architecture, grain attributes, tillers, spikes, and spikelets. Grain traits were obtained by scanning the grains to generate images, which were then analyzed using SC‐G software (Wanshen Detection Technology Co., Ltd., Hangzhou, China). To obtain plant architecture and tiller traits, individual plants were collected from the field, and images were captured against a black background with a scale marker for accurate measurements. Semantic segmentation was applied to isolate the main stem from the background, and an object detection algorithm was used to identify the positions of nodes along the stem. The wheat stem skeleton was extracted using the Zhang‐Suen thinning algorithm, refined with a depth‐first search (DFS) algorithm, and smoothed using the Savitzky‐Golay (SG) filter to enhance measurement accuracy. This pipeline allowed the determination of the main stem length, which was defined as plant height, and the calculation of internode lengths based on node positions. Stem width was measured by calculating the perpendicular distances from the skeleton to the stem boundary within a 2‐cm region below each node.^[^ ^50^ ^]^

To obtain spike and spikelet traits, images of wheat spikes were captured using the same imaging protocol used for plant imaging. A semantic segmentation network was applied to isolate the spikes from the background, enabling the calculation of basic phenotypic traits such as spike area and perimeter from the extracted masks. To measure spike length accurately, the masks were further refined using the DFS algorithm and smoothed with an SG filter. Each spike was divided into 20 equal slices along its length, and the width was measured at each slice. Additionally, instance segmentation was applied to identify fertile and infertile spikelets, allowing us to measure spikelet traits such as length, area, and angle relative to the main axis. Spikes were further divided into structural sections based on their length, and spikelet traits were analyzed within these regions.^[^ ^50^ ^]^ We provide a complete list and description of all the traits analyzed in this study in Table S1, Supporting Information.

### Analysis of Spikelet Geometric Traits

Each line has a different number of spikelets. R‐4.3.1^[^ ^51^ ^]^ was used to normalize the spikelet numbering system for each line, so that the spikelets at the same position are aligned to the same level. Then, a polynomial regression analysis was performed using the poly() function in R, and the resulting polynomial coefficient matrix was used for data fitting with the lm() function. The fitted curve was divided into 20 equal segments, resulting in interpolations at 21 nodes. The 21 points were sequentially grouped into four parts (3, 8, 8, and 2 points) according to the standard deviation of each interpolated point of the whole population, representing distinct spike regions (Figure S2f,g, Supporting Information).

### Permutation Testing to Determine the Filtering Threshold

The raw VCF file, obtained from SNP calling using the GATK best practice workflow in previous studies,^[^ ^20^ ^]^ was filtered with the criteria “%QUAL < 50 || INFO/DP < 5” of BCFtools v1.18^[^ ^52^ ^]^ resulting in 6 140 927 sites. To eliminate false‐positive mutation sites, permutation testing was used to determine the filtering threshold. The genotype information for each sample in the clean VCF file was randomly shuffled with respect to site position. The mutation frequency for each site in this randomized VCF file was calculated, and the lowest 500 mutation frequencies were selected as the result of a single permutation test. This permutation test was repeated 1000 times, generating a trend plot (Figure S4a, Supporting Information). The result ranked fifth, with a mutation frequency of 0.7%, was selected as the mutation frequency threshold for filtering (*p*‐value = 0.01). Sites with a mutation frequency greater than 0.7% were removed, leaving 4 228 167 mutation sites. Mutation effects were annotated and categorized into four functional classes (high, moderate, low, and modifier impact) using SnpEff (v5.2) with default parameters.

### GH‐GLA Framework

The concepts of forward genetics and reverse genetics were used as the basis for two analytical pathways. The forward approach involves grouping individuals based on the effects of mutation predicted by SnpEff at specific genes, and assessing whether significant phenotypic differences exist between these groups. For the reverse approach, the population was divided into two groups based on whether their phenotypes deviate from the population average before detecting whether mutation sites are significantly enriched in genes in one group. The goals of this computational framework are: 1) identify genes with enriched mutations in samples with prominent phenotypes; and 2) examine whether the phenotypes of samples with mutations in genes show significant changes. The results from these two approaches serve as mutual validation to ensure the reliability of the findings. The first approach uses a chi‐square (*χ* ^2^) test for association analysis:

(1) $$\chi^{2}=\sum \frac{{\left(Obs-Exp\right)}^{2}}{Exp}$$

where, for each gene, *Obs* represents the number of mutant lines with phenotypic differences from the wild type and 𝐸*xp* represents the number of mutant lines with mutations in that gene. The *χ* ^2^ value thus indicates the degree of deviation.

The second approach uses a linear regression model for association analysis:

(2) $$y=a\sum_{k=1}^{n}e+b$$

In this model, 𝑎 represents the number of mutation sites, *n* represents the number of samples, 𝑒 denotes the mutational effect of the mutations in the gene for a given sample, 𝑏 is the random error, and 𝑦 is the phenotypic data. Regression analysis was used to determine the association between genotype and phenotype. Alternatively, a mixed linear model can be used, where 𝑎 is defined as whether the gene contains a mutation (Figure S5a,b, Supporting Information).

Each approach will yield a *p*‐value reflecting the degree of significance of the association between each gene and phenotype. The top 300 most significant genes from each approach are selected, and the intersection of the gene sets identified by the two approaches is taken. The common genes are then added to the candidate gene pool. Permutation testing is performed 1000 times on both approaches to determine the significance threshold. Genes in the candidate gene pool with a significance greater than the threshold are considered candidate genes. As the expression levels of candidate genes across different tissues can serve as important selection criteria to help prioritize targets for functional validation, RNA‐seq data from multiple datasets were compiled and included in Table S5, Supporting Information.

### Construction of the Epistatic Interaction Network

Each gene was organized into a VCF format based on the presence or absence of mutations in the lines, before using MatrixEpistasis^[^ ^53^ ^]^ to calculate the epistatic interactions between gene pairs for each phenotype. The top 1000 most significant gene pairs for each phenotype were selected. Network visualization was performed using Gephi v0.10,^[^ ^54^ ^]^ and network properties were calculated using the R package igraph.^[^ ^55^ ^]^

### RNA‐Seq Data Processing and Gene Expression Analysis

RNA‐seq data processing and analysis were conducted following the methods described in Shi et al.,^[^ ^25^ ^]^ ensuring consistency and comparability with the previously published study. Detailed tissue types and developmental stages analyzed are provided in Table S12, Supporting Information. Differential gene expression levels were quantified as fragments per kilobase of transcript per million mapped reads (FPKM) values and visualized using appropriate bioinformatics tools.

### Phylogenetic Analysis

To perform phylogenetic analysis, the amino acid sequences of orthologs of TaAN‐1, TaBAM5L, and TaXTH28L from different plant species were downloaded from Ensembl Plants (http://plants.ensembl.org/index.html). Mega X^[^ ^56^ ^]^ was employed to reconstruct the phylogenetic tree with the following programs: first, all protein sequences were aligned in ClustalW with default parameters; second, the maximum likelihood method was used to build a phylogenetic tree with the Poisson model and 1000 bootstrap replicates.

### Haplotype Analysis of TaAN‐1‐2B, TaBAM5L‐5A and TaXTH28L‐4A

Haplotype analysis of *TaAN‐1‐2B*, *TaBAM5L‐5A*, and *TaXTH28L‐4A* was performed using natural variations identified by a whole‐exome sequencing project of 262 Chinese wheat mini‐core accessions.^[^ ^39^ ^]^ Variations were extracted from regions spanning 1 kb upstream of the transcription start site to 1 kb downstream of the transcription end site. These variations were analyzed using the geneHapR^[^ ^57^ ^]^ software to identify and evaluate.

Phenotypic data were obtained from the same years and locations as described in the previous study,^[^ ^45^ ^]^ ensuring consistency and comparability. The average values of the phenotypic traits were calculated and used for association analysis with the identified haplotypes.

### Statistical Analysis

Statistical analysis and visualization of both genotypic and phenomic data were performed using the R 4.3.1. This included quantification of mutation counts, calculation of phenotypic correlations, and cluster analysis. The experimental data were presented as mean ± standard deviation without additional preprocessing. Data visualization included scatter plots and bar graphs for presenting mean values with error bars representing SD. All analyses were performed with n = 5 independent replicates per group. Statistical significance was determined using two‐tailed t‐tests and one‐way ANOVA with significance levels denoted as *p < 0.05, **p < 0.01, ***p < 0.001, ****p < 0.0001, and n.s. for not significant. GraphPad Prism 9.0 was employed for generating scatter plots, bar graphs, heatmaps, and line graphs, as well as for performing ANOVA, and t‐tests.

### Data Availability

The raw and filtered VCF files by WES from this study were deposited to the Genome Variation Map^[^ ^58^ ^]^ under accession number GVM000963 and to the National Genomics Data Center,^[^ ^59^ ^]^ China National Center for Bioinformation/Beijing Institute of Genomics, Chinese Academy of Sciences, where they are publicly accessible at https://ngdc.cncb.ac.cn/gvm. The phenotypic data reported in this study were deposited to OMIX under accession number OMIX010498. The published RNA‐seq data from KN9204 used for analysis were downloaded from CNCB‐NGDC under BioProject number PRJCA004416.^[^ ^25^ ^]^

### Code Availability

All scripts and codes associated with this project are available via GitHub at https://github.com/gaze‐abyss/GH‐GLA.

## Conflict of Interest

The authors declare no conflict of interest.

## Author Contributions

H.W., F.S., Z.S., and Y.Y. contributed equally to this work. H.W. led the bioinformatics analysis and wrote the first version of the manuscript; F.S., S‐s.Z., and N.J. conducted image‐based phenotyping; Z.S. and Y.Y. performed molecular experiments; Y.D., T.Z., C.Z., J.X., and Y.Z. contributed to data collection and presentation of the results; W.T., J.L., X.L., S‐s.Z., and N.J. contributed to data interpretation and manuscript writing; F.H. and S.Z. conceived the study, designed the research, and edited the manuscript.

## Supporting information



Supplemental Table

Supporting Information

## Data Availability

The data that support the findings of this study are openly available in Genome Variation Map in National Genomics Data Center, China National Center for Bioinformation / Beijing Institute of Genomics, Chinese Academy of Sciences at https://bigd.big.ac.cn/gvm/getProjectFile?t=fb8b13bc, reference number 963.
