# Supplementary material for: A Forward Genetics Strategy for High‐Throughput Gene Identification via Precise Image‐Based Phenotyping of an Indexed EMS Mutant Library
Source: Adv Sci (Weinh). 2025 Sep 29;12(47):e14793. doi: 10.1002/advs.202514793 (PMC12713079; doi:10.1002/advs.202514793)
Supplement: Supplementary file 2 — Supporting Information [file ADVS-12-e14793-s001.docx]

**Figures S1-9**


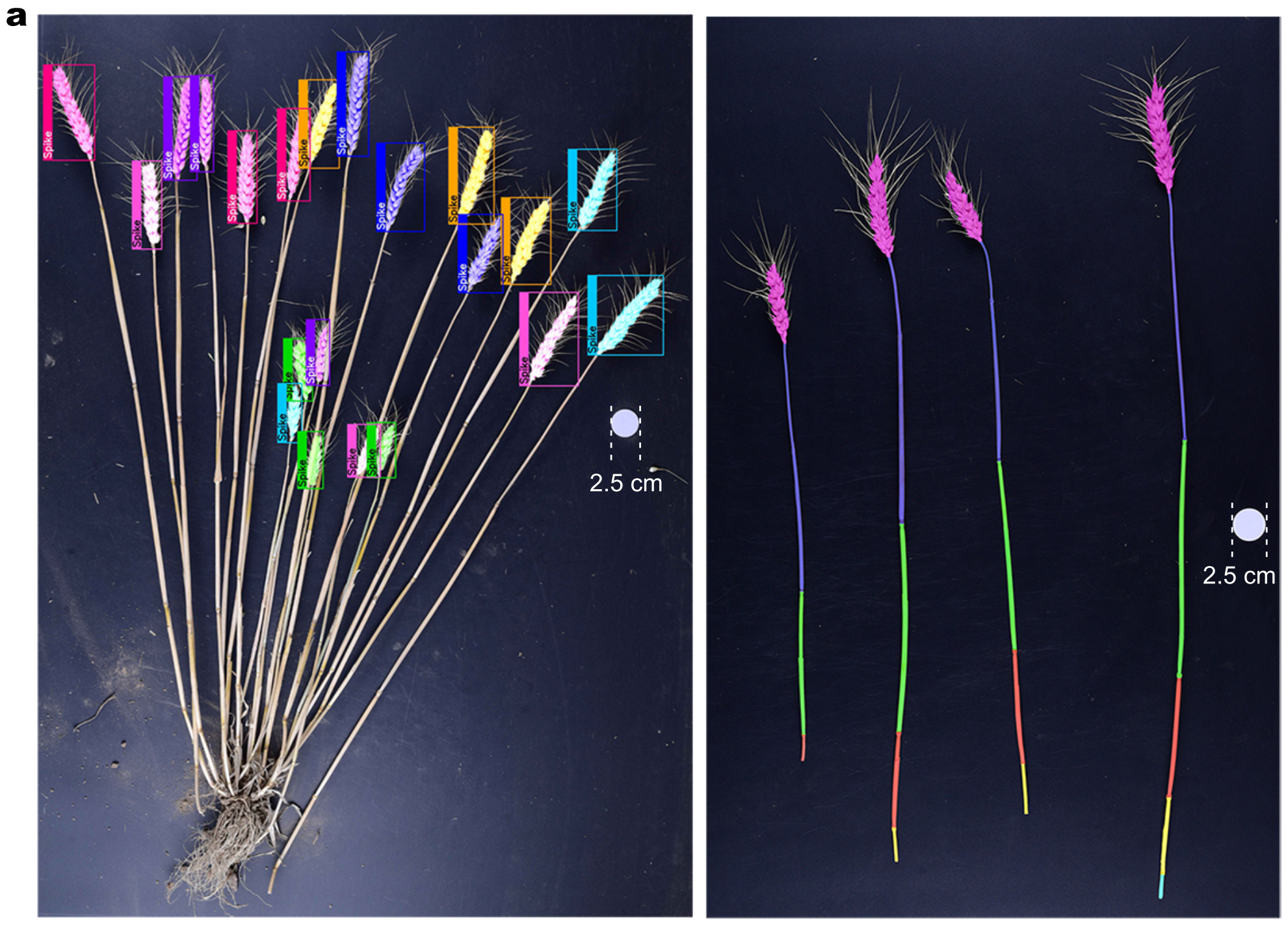


Figure. S1 Deep learning–based phenotype extraction and analysis in wheat.

**a)** Phenotyping of a wheat spike by instance segmentation for counting effective tiller number (left), and phenotyping of internode and plant height by segmentation of all internodes and spike on the main stem to calculate internode length and plant height (right). The white dot (diameter, 2.5 cm) indicates scale.


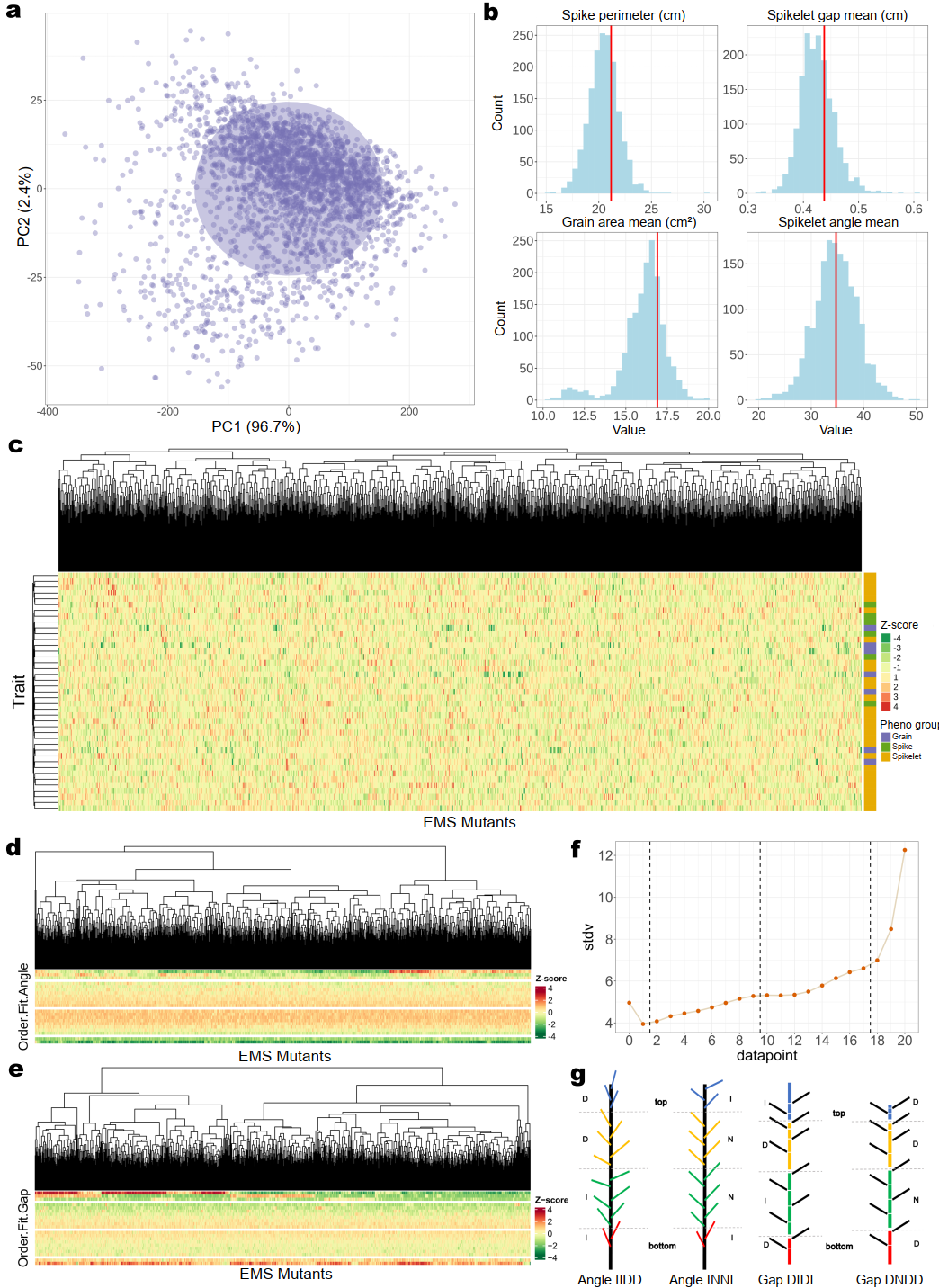


Figure. S2 Phenomics analysis and quantitative analysis of geometric traits.

**a)** Principal component analysis (PCA) plot of phenotypic variation in the natural population (PC1 = 96.7%, PC2 = 2.4%). **b)** Distribution of certain phenotypic values in the EMS population. The red line indicates the average value of the wild-type population, with most mutant values being left-shifted relative to the WT mean. **c)** Clustering of lines after randomly assigning normalized data for each of 41 phenotypes to a different line. No significant modular structure was detected. **d, e)** Clustering of lines after randomly assigning the fitted values of spikelet angle (**d**) and spikelet gap (**e**) to a different line. No significant modular subgroup was detected. **f)** Standard deviation of spikelet angle at different positions along the stem, which can be divided into four groups based on the trend. **g)** Diagram of different spike types defined by various spikelet geometric phenotypes.


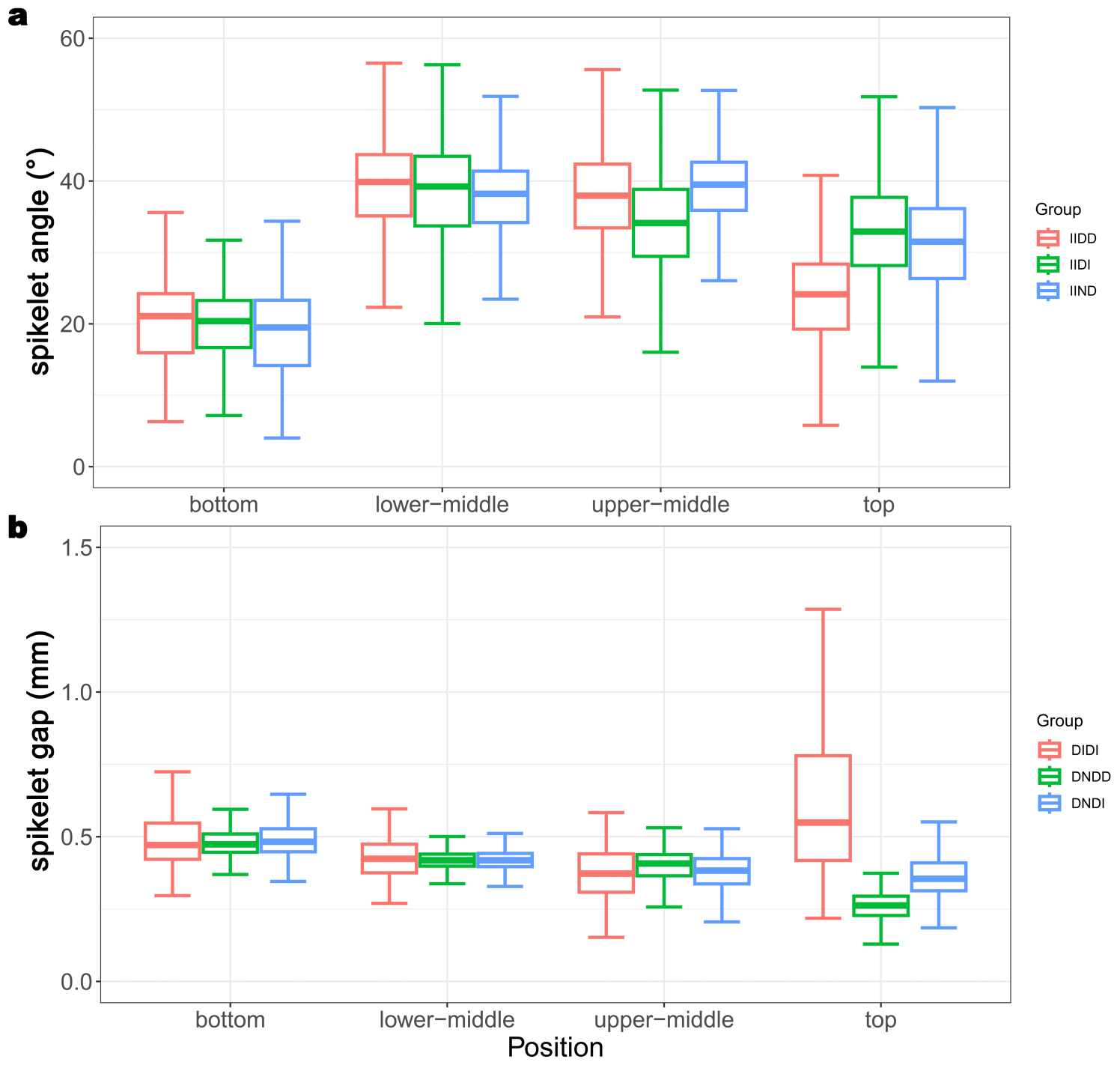


Figure. S3 Spikelet angle and gap measurements for three spike types in the KN9204 EMS mutant population.

**a)** Spikelet angle ranges among the three spike types. **b)** Spikelet gap ranges among the three spike types. The x-axis indicates the relative position of the spikelet along the spike: bottom, lower-middle, upper-middle, and top. Each box plot displays the median (center line), interquartile range (box), and minimum/maximum values excluding outliers (whiskers).


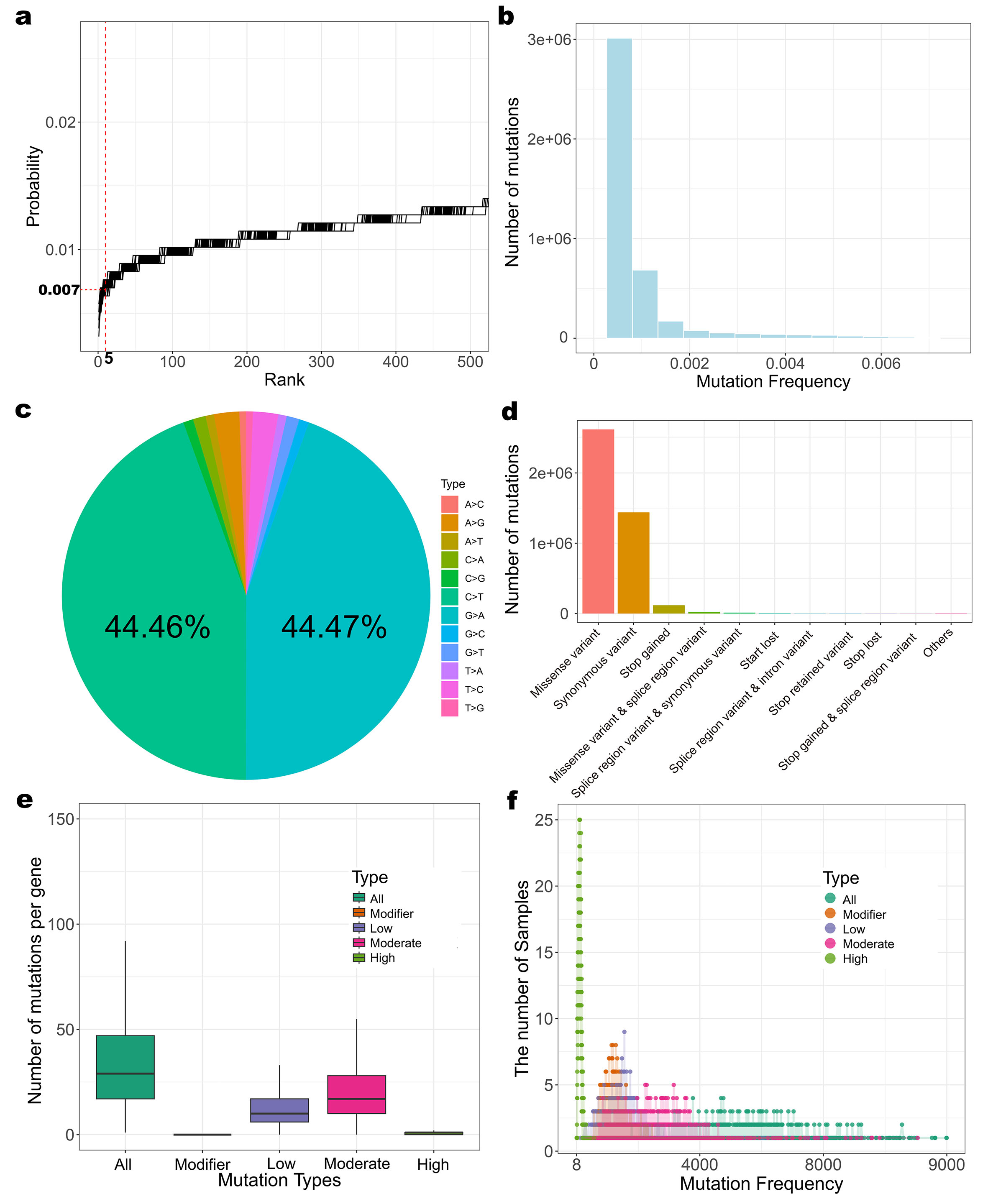


Figure. S4 Preparing a new genotyping dataset for association analysis.

**a)** Each line represents one simulation of the frequency of the mutation existing in the population. Plot of the 500 lowest probabilities from 1000 permutation tests. The vertical red line highlights the 5^th^ lowest probability. **b)** Distribution of the number of sites with different mutation frequencies. Most mutation sites have a mutation frequency below 0.1%. **c)** Proportions of types of nucleotide mutations in the updated genotyping dataset. EMS-type mutations (C-to-T and G-to-A) account for more than 89% of all mutations. **d)** Functional annotation of mutations in genes. **e)** Number of mutations with different effects in each gene. Each box plot displays the median (center line), interquartile range (box), and minimum/maximum values excluding outliers (whiskers). **f)** Number of mutations with different effects in each EMS line.


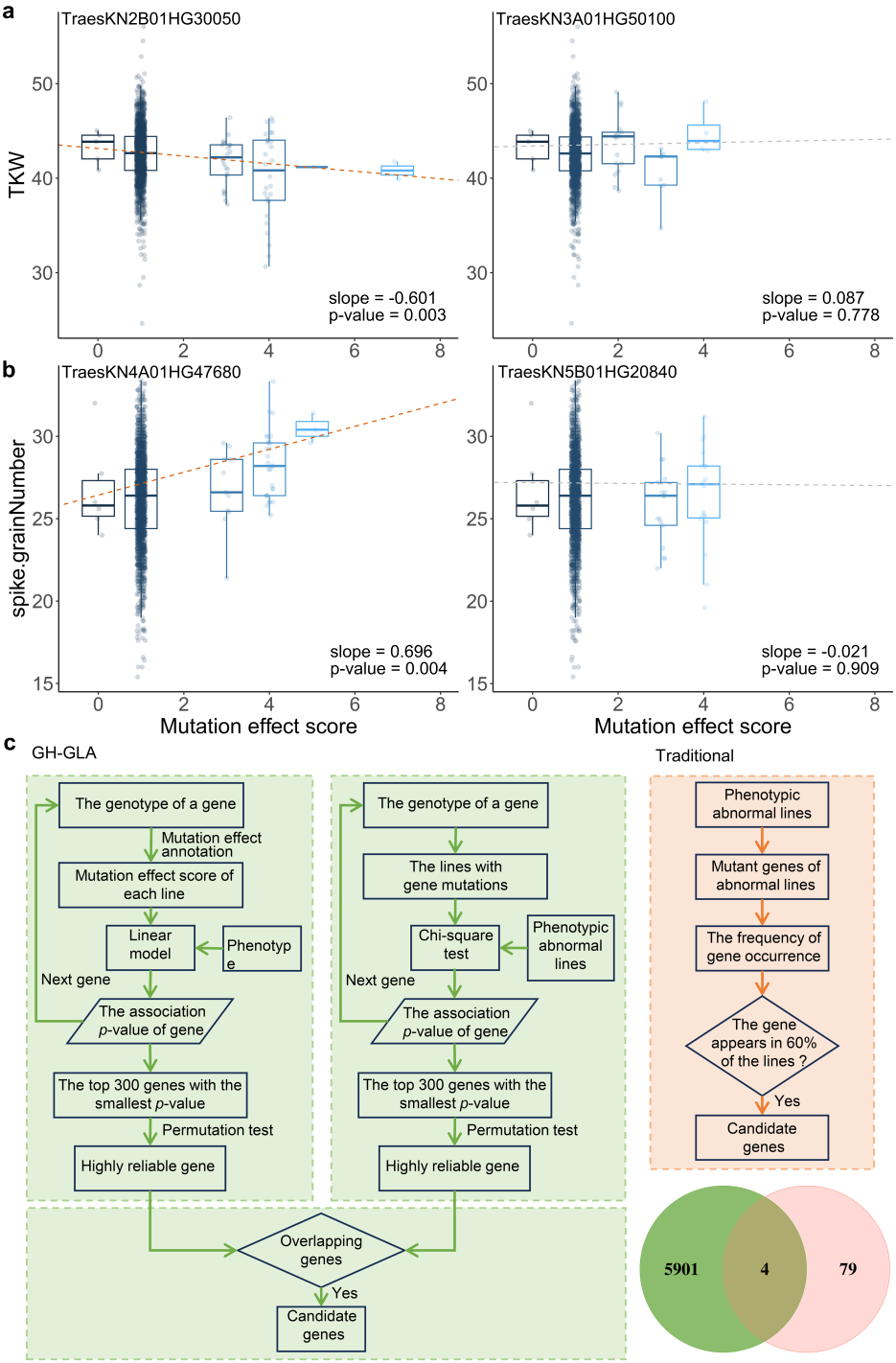


Figure. S5 Association analysis using a linear modeling approach.

**a, b)** Association of genotyping data with TKW (**a**) or number of spike grains (**b**) through linear modeling. Mutation effect scores were derived from the predicted effects of mutations within each gene in individual samples. Each dot represents an individual EMS line, grouped by mutation effect score. Each box plot represents the median (center line), interquartile range (box), and minimum/maximum values excluding outliers (whiskers). The red dashed lines represent a statistically significant regression slope (*P* < 0.01), and gray dashed lines indicate non-significant associations (*P* ≥ 0.05). **c)** Analysis workflows for the GH-GLA pipeline (left, green background) and the conventional EMS population–based mapping approach MutMap (right, red background).





Figure. S6 Candidate genes identified from gene-level association analysis.

**a)** Number of candidate genes for each phenotype in the association analysis. Traits are grouped by phenotypic categories—plant (blue) and tiller (pale yellow)—and genes are color-coded as homologous to functionally characterized genes (dark yellow) or to non-functionally characterized genes (green). **b, c)** Manhattan plots of the association results for grain number (GN, b) and spikelet gap (c). The red dashed lines indicate the significance threshold, which was determined by permutation test. **d–f)** GO term enrichment analysis of candidate genes related to all traits (**d**), grain-associated traits (**e**), and spike-associated traits (**f**). Symbol size represents the number of genes, and its color indicates statistical significance.





Figure. S7 Phenotypic variation in lines carrying mutations in candidate genes

**a–c)** Boxplots showing the number of grains per spike (**a**), spike length (**b**), and spikelet number (**c**) in lines carrying mutations in the candidate gene and other EMS lines. Red indicates lines with mutations in the candidate gene; blue indicated lines without a mutation in the candidate gene. Each box plot represents the median (center line), interquartile range (box), and minimum/maximum values excluding outliers (whiskers). A *t*-test was used to determine the statistical significance of differences. ****, *P* < 0.0001. Representative photographs of the seed heads from these lines are shown on the right. Scale bars, 1 cm.


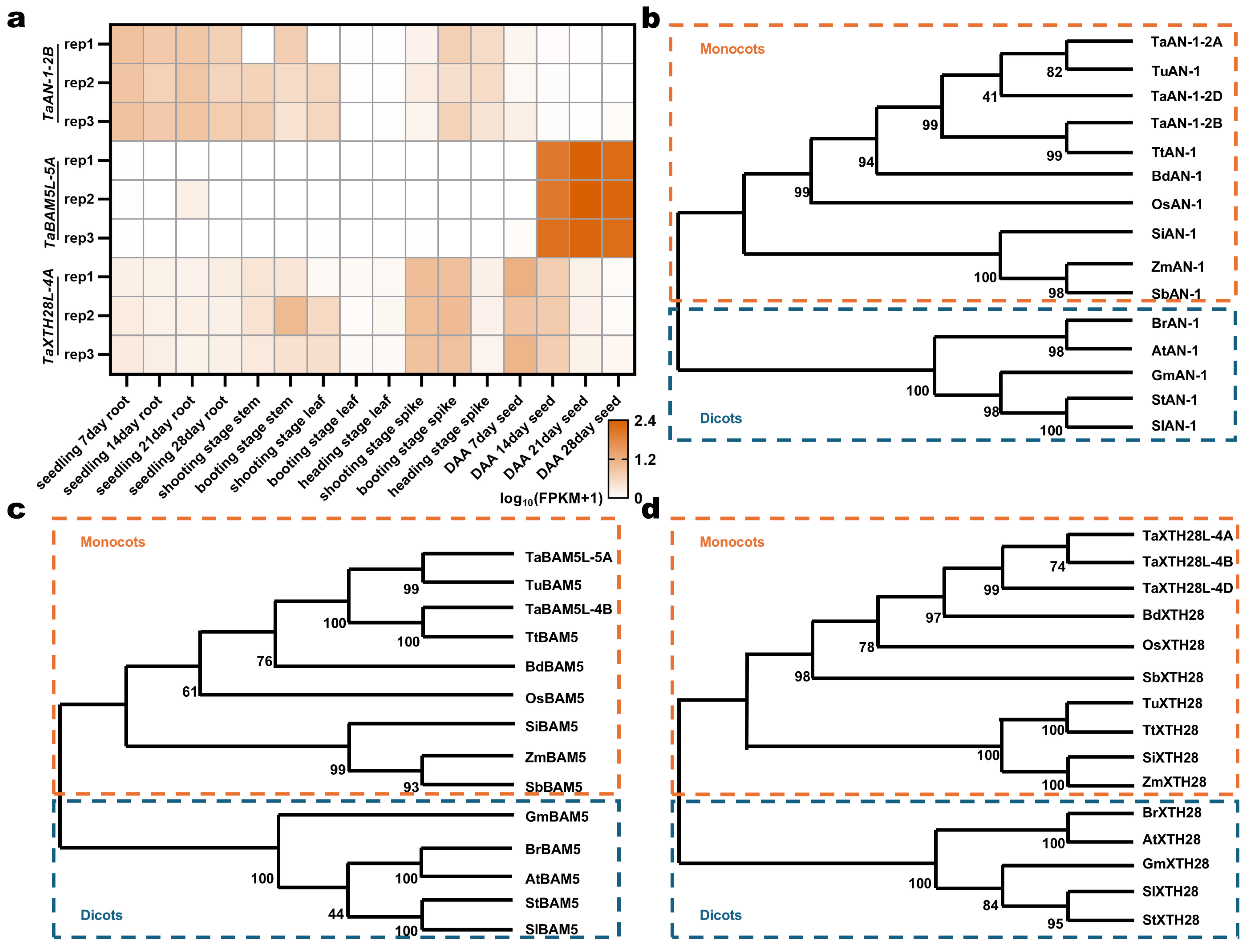


Figure. S8 Expression patterns and phylogenetic analysis of candidate genes

**a)** Spatiotemporal expression analysis of *TaAN-1-2B*, *TaBAM5L-5A*, and *TaXTH28L-4A* in cv. Kenong9204 (KN9204) based on published RNA-seq data. The color scale shows log_10_(FPKM). **b–d)** Phylogenetic analysis of AN-1, BAM5, and XTH28 from *Arabidopsis thaliana* (*At*), tomato (*Solanum lycopersicum*, *Sl*), soybean (*Glycine max*, *Gm*), sorghum (*Sorghum bicolor*, *Sb*), maize (*Zea mays*, *Zm*), rice (*Oryza sativa*, *Os*), *Brachypodium distachyon* (*Bd*), potato (*Solanum tuberosum*, *St*), *Setaria italica* (*Si*), bread wheat (*Triticum aestivum*, *Ta*), *Triticum urartu* (*Tu*), *Triticum turgidum* (*Tt*), and field mustard (*Brassica rapa*, *Br*). Bootstraps, 1,000. The dashed boxes outline the proteins from dicotyledonous (blue) and monocotyledonous species, (orange).


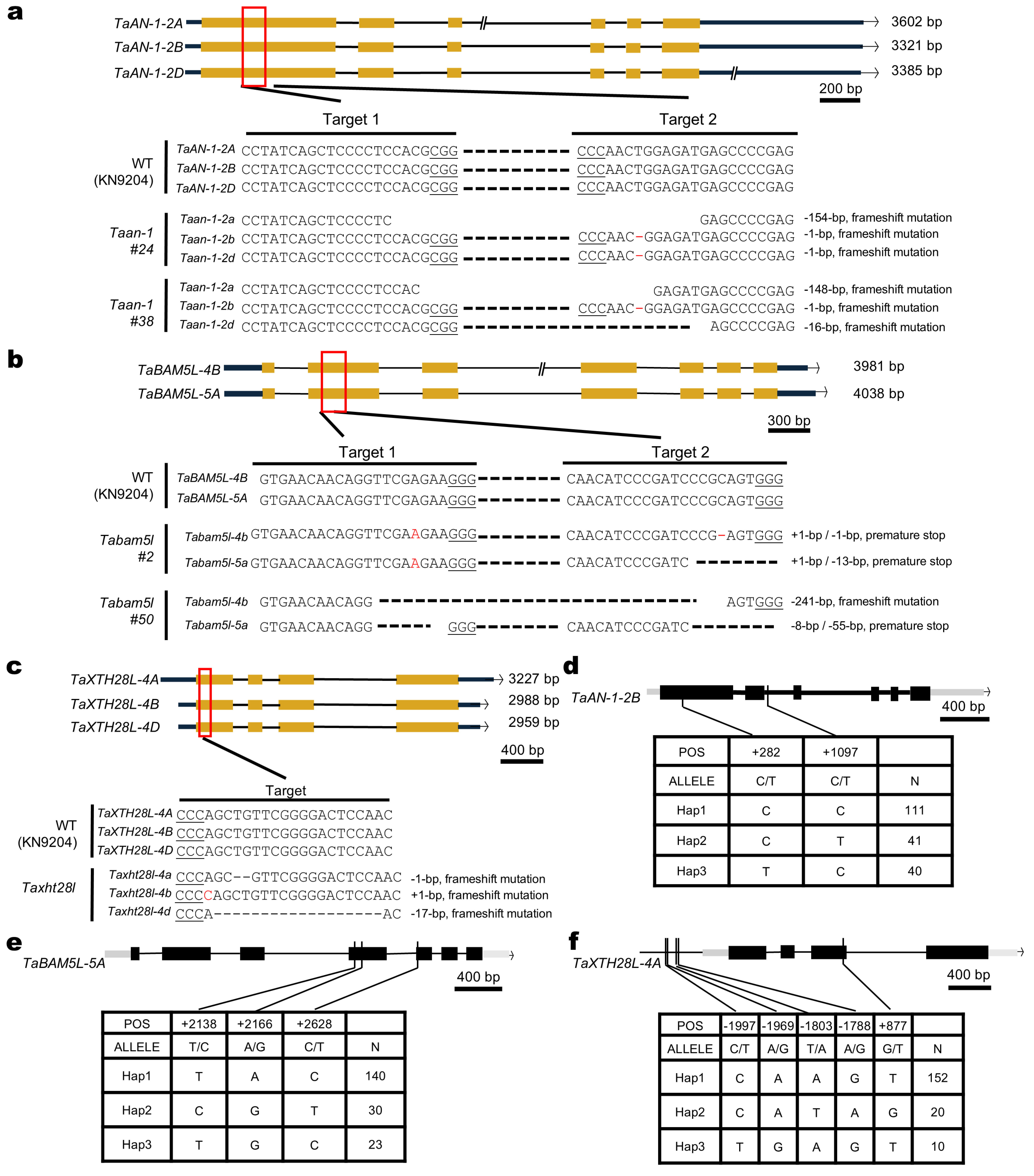


Figure. S9 Characterization of gene-edited mutants and analysis of natural variation in candidate genes

**a–c)** Target sites for the single guide RNAs (sgRNAs) used to generate the *Taan-1*, *Tabam5l*, and *Taxth28l* mutant lines using CRISPR/Cas9. **d–f)** Diagram of the *TaAN-1-2B*, *TaBAM5L-5A*, and *TaXTH28L-4A* loci (top), showing the polymorphisms distinguishing the three haplotypes in the common wheat population.

**Tables S1–12**

Table S1. Phenotypes of the i-traits generated in this study.

Table S2. Pearson correlation coefficients between all pairs of phenotypes.

Table S3. Basic statistics of the phenotypes for wild-type plants and the EMS panel.

Table S4. Candidate genes predicted using traditional methods.

Table S5. Candidate genes predicted using GH-GLA.

Table S6. FPKM values from RNA-seq.

Table S7. Genes analyzed and discussed in this study.

Table S8. Epistatic gene pairs detected for each trait.

Table S9. Properties of the epistatic network for each trait.

Table S10. Gene ontology term enrichment analysis for network modules.

Table S11. Primers and sequences used for molecular experiments.

Table S12. List of samples used to generate RNA-seq data.
